# Intrinsically Motivated Exploration of Learned Goal Spaces

**DOI:** 10.3389/fnbot.2020.555271

**Published:** 2021-01-12

**Authors:** Adrien Laversanne-Finot, Alexandre Péré, Pierre-Yves Oudeyer

**Affiliations:** Inria, Univ. Bordeaux and Ensta ParisTech, Paris, France

**Keywords:** sensorimotor development, unsupervised learning, representation learning, goal space learning, intrinsic motivation, goal exploration

## Abstract

Finding algorithms that allow agents to discover a wide variety of skills efficiently and autonomously, remains a challenge of Artificial Intelligence. Intrinsically Motivated Goal Exploration Processes (IMGEPs) have been shown to enable real world robots to learn repertoires of policies producing a wide range of diverse effects. They work by enabling agents to autonomously sample goals that they then try to achieve. In practice, this strategy leads to an efficient exploration of complex environments with high-dimensional continuous actions. Until recently, it was necessary to provide the agents with an engineered goal space containing relevant features of the environment. In this article we show that the goal space can be learned using deep representation learning algorithms, effectively reducing the burden of designing goal spaces. Our results pave the way to autonomous learning agents that are able to autonomously build a representation of the world and use this representation to explore the world efficiently. We present experiments in two environments using population-based IMGEPs. The first experiments are performed on a simple, yet challenging, simulated environment. Then, another set of experiments tests the applicability of those principles on a real-world robotic setup, where a 6-joint robotic arm learns to manipulate a ball inside an arena, by choosing goals in a space learned from its past experience.

## 1. Introduction

Although (deep) reinforcement learning has seen great successes in the recent years, designing learning algorithms remains challenging for many tasks. For example, it can be quite difficult to specify the task in terms of reward (Christiano et al., 2017) or learning the task may require mastering many intermediate steps (Bengio et al., 2009). An extreme case being when the experimenter does not know what is achievable. For example, this can happen if we consider a robot placed in an environment that contains different objects. In many cases, even though the dynamic is known, one does not know *a priori* the whole range of possible behaviors. The robot might be able to grasp some objects but is unable to grasp others. This is also the case for cellular automata like the game of life. In such a game, the “experimenter” can be thought of as an agent that performs experiments, and the initial conditions of the experiment correspond to the action. In such a game, the range of possible behavior is very hard to characterize, and so designing a reward is not feasible as there is no clear objective nor a measurable quantity to optimize (Reinke et al., 2020). One may also argue that a similar scenario is at play for children: they do not know the extent of the possible behaviors that they can or will be eventually able to produce.

In contrast, children are capable of learning a wide variety of skills by themselves, purely driven by their curiosity. Crucially, infants are capable of setting goals for themselves for tasks that they wish to achieve or want to learn (Berlyne, 1966; Gopnik et al., 2009). Without any external supervision, they are capable of designing their own curriculum by selecting tasks that they regard as interesting among all the possibilities offered by the environment. This observation led, in the context of developmental robotics (Cangelosi and Schlesinger, 2018), to the development of an approach based on modeling the effects of intrinsic motivations to improve the exploration capabilities of artificial agents (Oudeyer et al., 2007; Baldassarre and Mirolli, 2013). The general idea is to design efficient curiosity mechanisms that allow the agent to explore its environment and to discover what outcomes can be produced in the environment autonomously, without any specific instructions or reward. Providing efficient solutions to this problem is one of the key challenges in lifelong learning.

Developing machine learning systems that are capable, without supervision, to explore efficiently by self-generating goals and an associated curriculum is of paramount importance in order to solve complex tasks that involve long time horizon goals. Such approaches would dramatically reduce the burden of designing specific *ad-hoc* rewards for each task (Christiano et al., 2017). It would also reduce the need for a hand-crafted curriculum which can be hard to design in some environments (Schmidhuber, 1991; Oudeyer et al., 2007; Graves et al., 2017). Overall, it would compensate for the failure of deep reinforcement algorithms on difficult exploration problems.

Discovering autonomously what outcomes can be produced by performing actions in an environment can be highly useful for a learning agent. This knowledge can then be used to learn world models and repertoires of parameterized skills (Baranes and Oudeyer, 2013; Da Silva et al., 2014; Hester and Stone, 2017) which can then be used by a high-level policy. Discovering multiple ways to manipulate an object or perform a certain skill can also help quickly repair strategies in case of damages (Cully et al., 2015). Such an approach is not orthogonal to deep reinforcement learning techniques and one can envision schemes that combine both approaches. For example, it is possible to use the data collected by an autonomous agent to efficiently bootstrap exploration for deep reinforcement learning problems with rare or deceptive rewards (Colas et al., 2018; Conti et al., 2018) or to use intrinsic rewards designed in the context of autonomous learning to guide exploration of deep reinforcement learning agents (Pathak et al., 2017).

One approach that was shown to be efficient for autonomous exploration is known as Intrinsically Motivated Goal Exploration Processes (IMGEPs) (Baranes and Oudeyer, 2010; Forestier et al., 2017). This architecture is closely related to Goal Babbling (Rolf et al., 2010). In the IMGEPs framework, agents are equipped with a goal space. In general, this goal space represents the set of outcomes that can be produced by the learning agent. For example, for a robot whose purpose is to move a ball, the goal space could be the position or the trajectory of the ball. During the exploration, the agent will sample goals in this goal space before trying to reach them using a goal-conditioned policy. This goal-conditioned policy can be learned either using standard techniques for policy learning or using more involved reinforcement learning techniques when the environment requires it (Andrychowicz et al., 2017). In order to improve its goal policy, after selecting a goal, the agent will dedicate a budget of experiments to improve its performance regarding this particular goal. In practice the agent will often fail to reach its goal but will often discover new possibilities when trying a particular goal. In order to leverage these discoveries IMGEPs are often implemented using a population approach where the agent stores an archive of all the policy parameters and their corresponding outcomes. Storing outcomes together with the associated policy parameters allows the agent to learn in hindsight how to achieve each outcome he discovers, should he later sample it as a goal. Although goal exploration algorithms leverage goal policies to explore efficiently, we would like to stress that our objective is *not* to learn a forward or inverse model of the environment. Still, having access to a database with a wide range of diverse examples can often be leveraged to learn skills efficiently (Colas et al., 2018; Lynch et al., 2020).

Starting from this simple idea, it is possible to design multiple architectures that select goals according to different prioritization mechanisms. In particular, a powerful strategy is to select goals that maximize the empirical competence progress using multi-armed bandits (Baranes and Oudeyer, 2010). Using such a strategy one can observe that the learning agent will autonomously follow a learning curriculum where the agent progressively switches from simple to more complex goals while avoiding goals that cannot be achieved (Forestier et al., 2017). This strategy is highly efficient since the learning agent always targets goals that are neither too simple nor too complex. Even in their simplest form, where goals are selected randomly among all the possible goals, IMGEPs have been shown to provide good exploration performances. In practice, IMGEPs have been shown to enable high-dimensional robots to learn locomotion skills such as (Baranes and Oudeyer, 2013) manipulation of soft objects (Rolf et al., 2010; Nguyen and Oudeyer, 2014) or tool use (Forestier et al., 2017) efficiently. IMGEPs have also been shown to enable the autonomous discovery of patterns in a continuous game of life (Reinke et al., 2020).

A lot of work using goal architectures has been done on environments where the agent had access to high level representations of the world. For example, experiments performed in Baranes and Oudeyer (2013), Forestier et al. (2017), Florensa et al. (2017), and Andrychowicz et al. (2017) were performed on environments where the agent had direct access to the position, speed, or trajectory of the objects/bodies. In many cases such a representation is not available, and the agent only has access to low level perceptual measures such as pixels. While theoretically possible, designing an algorithm that extracts such features from the environment is often difficult and time consuming. Also, having to design a goal space greatly limits the autonomy of the agent as for each new environment one needs to design a specific goal space and develop ways to extract features associated to this goal space. An appealing alternative is to design exploration algorithms capable of learning and using a goal space directly from low perceptual measures.

Intrinsically motivated goal exploration algorithms are designed to autonomously discover the widest range of possible diverse effects that can be produced in an initially unknown environment. Thus, exploration algorithms are often evaluated using the coverage of the state space. Such measures were also used in a similar context for population-based divergent search algorithms (Cully, 2019; Paolo et al., 2020).

In this paper we study how goal exploration processes can be combined with goal space learning. In order to apply the ideas developed in Péré et al. (2018) to a real-world robotic setup with limited computing resources (raspberry Pi 3), we focus on population based IMGEPs. Our results show that representation learning algorithms can be efficiently combined with goal exploration algorithms to explore unknown environments. Experiments are performed on a real-world robotic setup where a 6-joint robotic arm learns how to manipulate a ball placed inside an arena. Overall, our results show that using a learned representation as a goal space leads to a better exploration of the environment than a strong baseline consisting of randomly sampling dynamic motion primitives^1^.

## 2. Intrinsically Motivated Goal Exploration Processes

**Figure 1:**
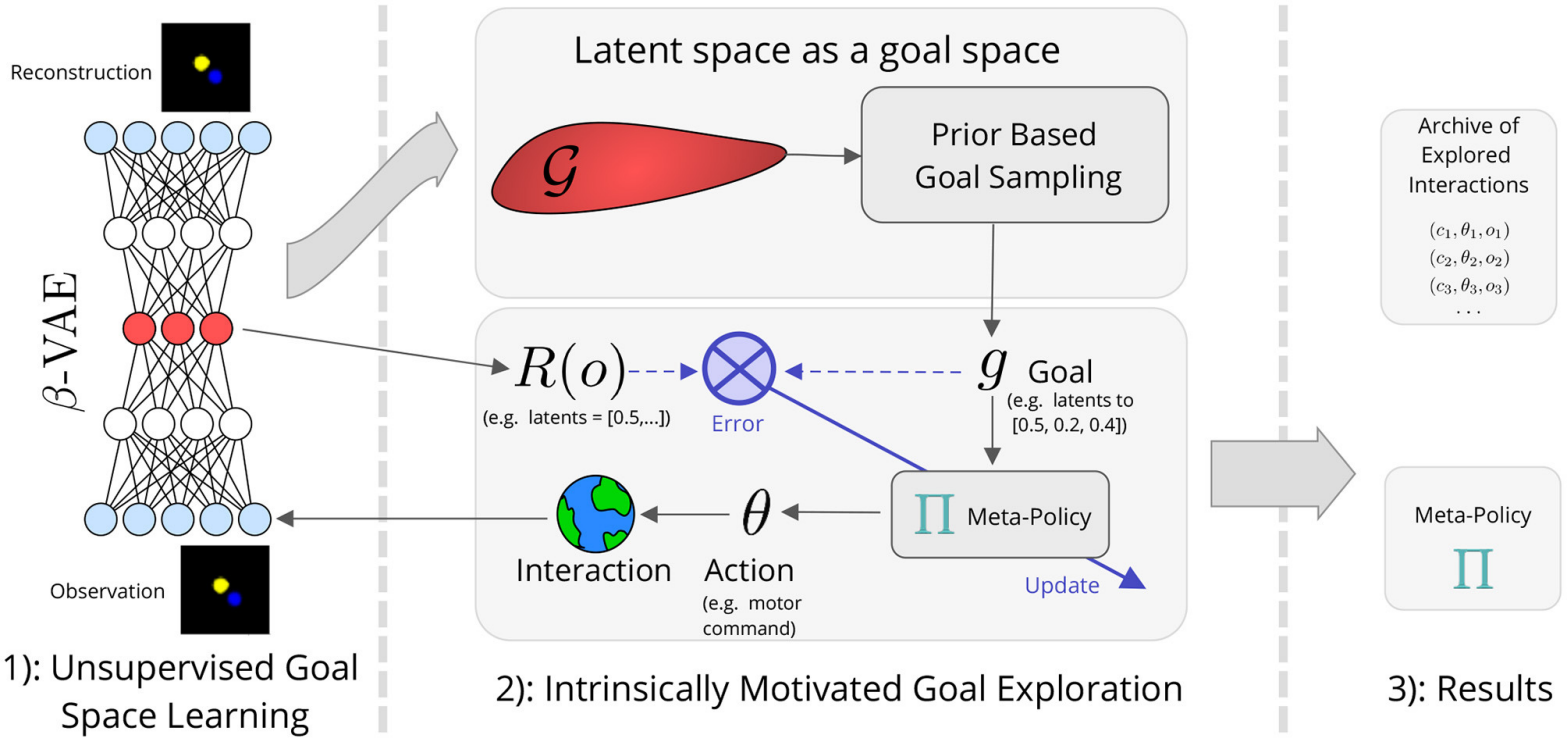
Intrinsically motivated goal exploration processes with unsupervised goal space learning (IMGEP-UGL).

This section details our approach that combines goal space learning with *Intrinsically Motivated Goal Exploration Processes* (IMGEPs). After introducing the exploration problem, we present a general version of IMGEPs. To better grasp the principles behind IMGEPs we first introduce IMGEPs using handcrafted goal spaces. We then present a method for learning the goal space that is used by IMGEPs. Finally, we present our architecture which combines goal space learning and IMGEPs. The overall architecture is summarized in Figure 1.

### 2.1. Intrinsically Motivated Goal Exploration Processes With Engineered Goal Spaces

#### 2.1.1. The Exploration Problem

Intuitively, an agent that explores well is, given an initially unknown environment, capable of discovering many possible behaviors, and ultimately, of generating a wide diversity of outcomes. A learning agent follows some kind of internal policy. This policy, together with the environment, determines the amount of diversity that the agent can produce. In practice, to explore efficiently an environment, the agent should gather information on the dynamic of the environment and leverage it to generate new outcomes (e.g., try to use tools that it already masters to generate new behaviors). For example, if the environment contains a ball and the agent wants to discover how this ball can be manipulated, it is necessary to use previous knowledge on how to catch the ball before trying to place it somewhere else.

Next, we introduce more formally the exploration problem and the IMGEP solution. Given an environment E, we define the following elements:

- A context space C. The context *c* represents the initial state of the environment. It corresponds to parameters of the experiment that are not chosen by the agent.
- A control (or parameterization) space Θ. θ ∈ Θ corresponds to the parameters of the experiment that the agent can control at will. For example, it corresponds to the trajectory of motor commands that the robot will use during one episode.
- an observation space O. Here we consider an observation *o* to be a vector representing all the signals captured by the agent sensors during an experiment (e.g., raw images).
- an environment dynamic D:C×Θ→O which maps parameters performed in a certain context, to observations (or outcomes). The dynamic is considered unknown to the agent.

**Figure 2:**
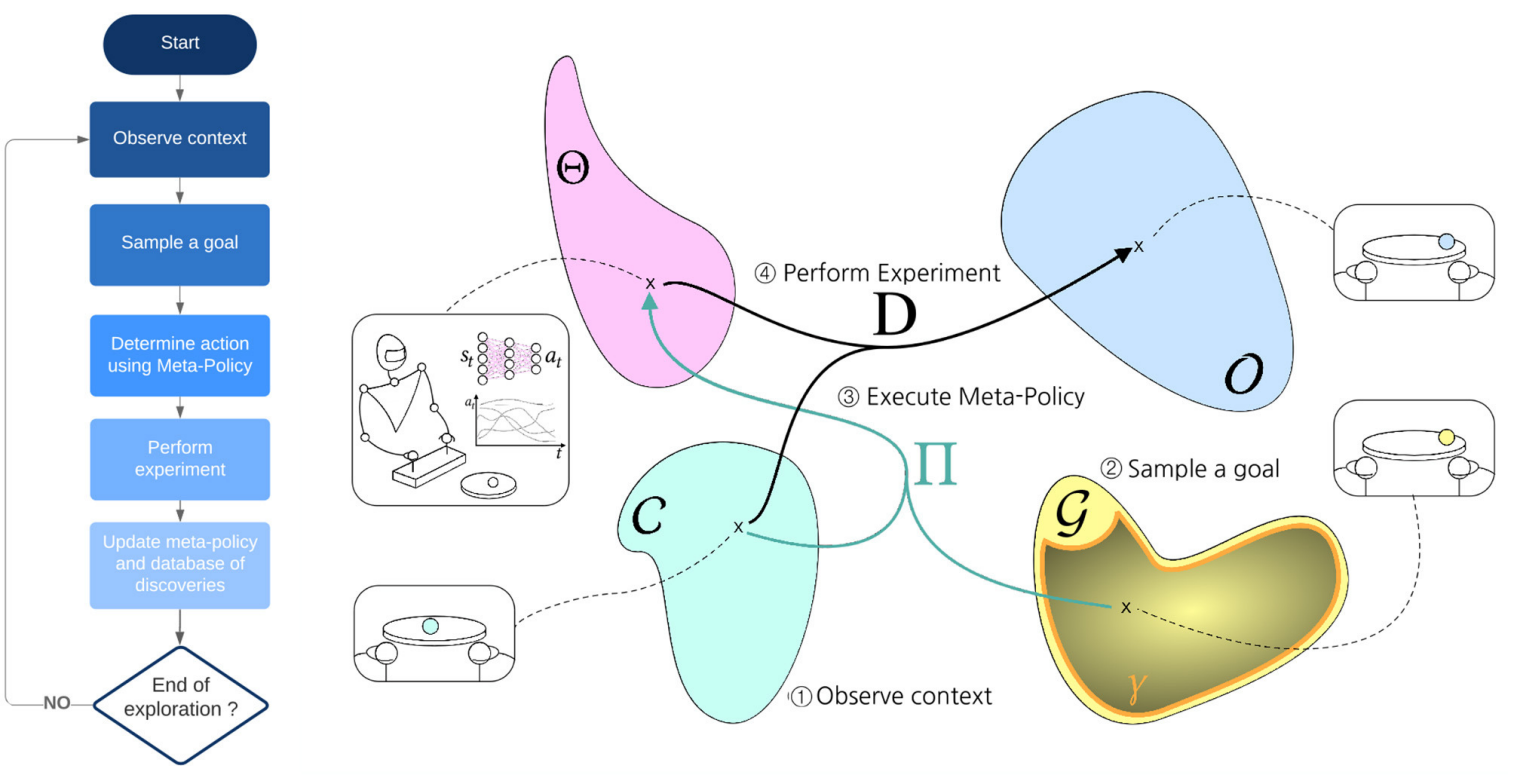
High-level view of intrinsically motivated goal exploration process.

For instance, as presented in Figure 2, a set of parameters could be the weights of a closed-loop neural network controller for a robot manipulating a ball. A context could be the initial position of the ball and an observation could be the position of the ball at the end of a fixed duration experiment. The exploration problem can then be simply put as:

Given a budget of *n* experiments to perform, how to gather tuples {(*c*_*i*_, θ_*i*_, *o*_*i*_)}_*i* = 1...*n*_ which maximize the diversity of the set of observations {*o*_*i*_}_*i* = 1...*n*_.

Quantifying the diversity of observation might cover different meanings depending on the context. In particular, consider the case where the environment contains several objects or offers various possible affordances. Manipulating the most complex objects often requires mastering easier sub-tasks (e.g., learn to move the robotic arm to control the position of the ball). Thus, exploration algorithms are often evaluated by measuring the diversity of outcomes obtained in the state space of the most complex task.

#### 2.1.2. Intrinsically Motivated Goal Exploration Processes

One approach that was shown to produce good exploration performances is Intrinsically Motivated Goal Exploration Processes (IMGEPs). A high-level view of IMGEPs is depicted in Figure 2. From a high-level perspective, IMGEPs follow this general scheme:

1. At each learning iteration the agent samples a goal from a goal space.
2. The agent observes the current state of the environment or context.
3. A meta-policy is then used by the agent to guess the best set of parameters to achieve the chosen goal, given the current context. This set of parameters (possibly with some exploration noise) is then used to perform an experiment.
4. The outcome obtained from the experiment is used to update the meta-policy. Note that information acquired when targeting a goal is used for improving the solution to other goals.

For example, in an environment where the learning agent is a robotic arm interacting with a ball, a goal can be the trajectory or end position of the ball after interacting for a certain duration.

We now introduce IMGEPs more formally. In order to introduce IMGEPs more formally we define the following elements:

- A goal space G. The elements g∈G represent the goals that the agent can set for himself.
- A goal sampling policy $\gamma :G\to {\mathbb{R}}^{+}$. The goal sampling policy is a probability distribution over the goal space. During exploration, the agent uses this distribution to sample goals in the goal space.
- A fitness function f:G×O→ℝ, internally used by the goal policy during training. This fitness function outputs the fitness of an observation for a given goal *g*.
- A meta-policy Π:G×C→Θ, which given a goal and a context, outputs a set of parameters that are most likely to produce an observation *fulfilling* the goal, under the current knowledge. The meta-policy can be learned using standard techniques from the set of actions and corresponding observations.

Using this framework, we introduce the Random Goal Exploration algorithm (Algorithm 1). It corresponds to an intrinsically motivated agent: at each exploration step the learning agent sets for himself his own goals and then tries to reach his goals using the goal policy. As described in Algorithm 1, in practice, the exploration often starts with some initial *Random Parameterization Exploration* iterations in order to initialize the meta-policy.

**Algorithm 1:**
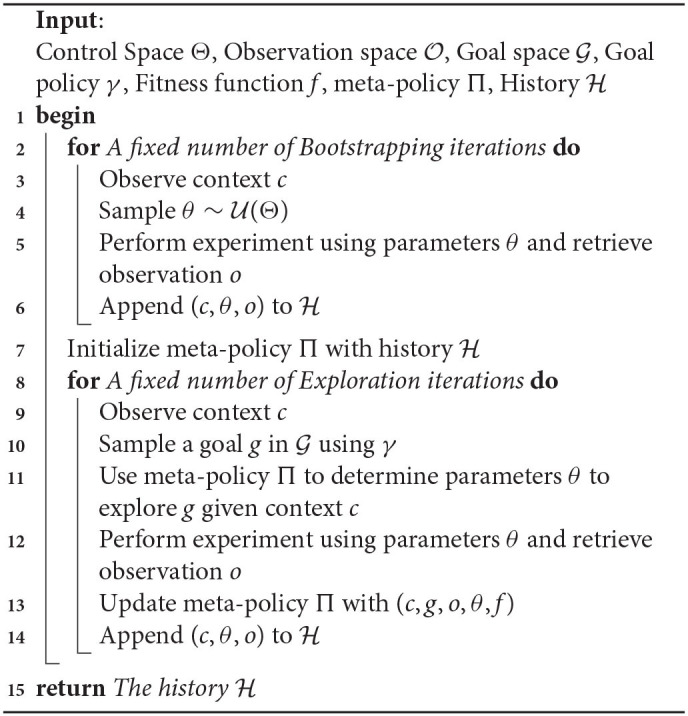
Random Goal Exploration with Engineered Goal Space (RGE-EFR).

Algorithm 1 can be instantiated into many particular algorithms sharing the same underlying principles. In particular, different goal policy mechanisms can be used (Baranes and Oudeyer, 2013; Forestier and Oudeyer, 2016). In our case the meta-policy is implemented using a nearest neighbor regressor. The inferred motor parameters are slightly perturbed using a Gaussian noise. This simple meta-policy was chosen because it is computationally efficient, and the robotic platform has low computing resources. For more complex environments it is possible to use state of the art deep reinforcement learning techniques (Andrychowicz et al., 2017; Warde-Farley et al., 2019). In general, it was observed that the exploration performances increase with the quality of the meta-policy (Forestier and Oudeyer, 2016). More details on our particular implementation and a detailed explanation of the meta-policy implementation are given in the Supplementary Material.

One might consider different goal sampling schemes depending on the environment. For example, in an environment that contains several goals that cannot be realized one might want to prioritize sampling goals that are possible to achieve. This can be done by sampling goals for which the agent is making progress in achieving them (Forestier and Oudeyer, 2016; Forestier et al., 2017). In practice, the goal sampling function γ is updated so that it tracks the learning progress associated to each goal. In other cases, in order to help bootstrap learning of newly discovered behaviors, one might want to encourage sampling goals which generally give rise to new discovery, similar to what is done in Quality-Diversity approaches (Lehman and Stanley, 2011b; Mouret and Clune, 2015; Cully and Demiris, 2017). This could be done by updating the goal sampling function γ in order to prioritize sampling goals that lead to a new discovery. This would encourage the agent to explore newly discovered behaviors even if it is not making any progress at controlling this behavior as it can take a long time before making any progress. Lastly, if the environment contains multiple objects, an efficient strategy for exploration is to divide the goal space into multiple sub-goal spaces, one for each object. This technique has been shown to automatically generate a learning curriculum (Forestier and Oudeyer, 2016; Forestier et al., 2017) and can also be adapted to learned goal spaces (Laversanne-Finot et al., 2018).

### 2.2. Goal Exploration With Learned Goal Spaces

#### 2.2.1. Learning a Goal Space

As mentioned in the introduction, often environments do not have associated goal spaces. One can always use the sensory space or observation space as a goal space, but in many cases the observation space does not make a good goal space. This is in particular the case when the agent perceives the world through low level perceptual measures such as images. In such a scenario, sampling a goal would require sampling an image. However, images with similar high-level properties, such as object positions, can be very different due to low level properties such as noise. One could also engineer a goal space from the sensory space. But in many cases handcrafting such a goal space from the sensory space is not easy. There are many cases in which we know the task that we want the agent to achieve but specifying it computationally can be difficult (Christiano et al., 2017). For instance, reaching the goal might not correspond to a readily available quantity, or there may be many different ways to achieve the task and it could be hard to find an objective that encompasses all the possible solutions.

As such one appealing alternative when a satisfactory goal space is not provided is to learn the goal space from observations of the environment. Ideally, to make the approach as simple and as general as possible, such a goal space should be learned from observations gathered by the agent during exploration.

A goal space is composed of several elements. In order to perform goal exploration using the learned goal space, it is necessary for the agent to be able to sample goals from the goal space. This ability takes the form of a probability function over the goal space. Also, in order to learn a policy, it is necessary that the agent be able to evaluate its performances in reaching goals. This ability is provided in the form of a fitness function over the goal space.

#### 2.2.2. Unsupervised Goal-Space Learning for IMGEP

In the following we detail a general approach, developed in Péré et al. (2018), for *Unsupervised Goal-space Learning* (UGL). The high-level idea is to learn a low-level representation of the environment that encapsulates and preserves the high-level information. Such a representation will then be used as the goal space for the agent. From the point of view of the agent, everything then works for the agent as if he is acting in a new environment given by the representation: instead of gathering raw observations the agent obtains encodings of those observations and learns a policy on top of these encodings.

In order to learn a goal space, the agent must first be provided or a set of observations {*o*_*i*_} that contain examples of outcomes that can be obtained in the environment must be gathered. This set of observations is then used to learn a low-dimensional representation using a representation learning algorithm. A wide range of representation learning algorithms can be used to learn the goal space (Péré et al., 2018). In this work we focus on Variational Autoencoders (VAEs). One of the advantages of VAEs is that they learn both a latent representation and a prior over the learned latent space. Deep representation algorithms are also known to handle better outliers.

The goal space learning procedure is described in Algorithm 2. Since representations learned with VAEs come with a prior over the latent variables, instead of estimating the goal-policies γ, we used the Gaussian prior assumed during training. Finally, the fitness function *f* is defined as the opposite of the distance between the goal and the latent representation of the observation: *f*(*g, o*) = −||*R*(*o*) − *g*||.

**Algorithm 2:**
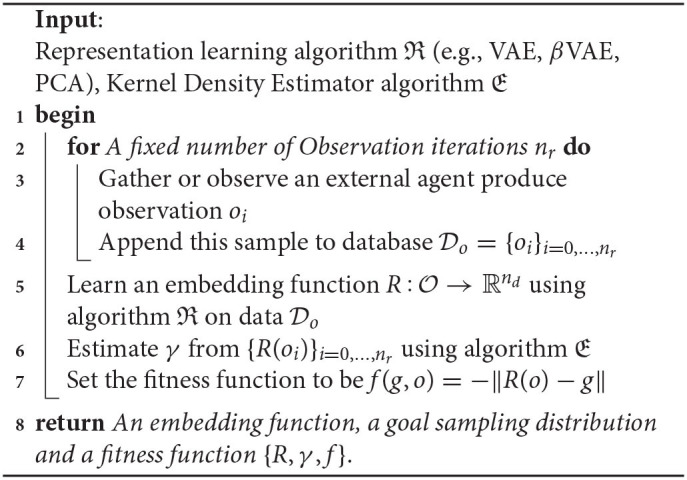
Unsupervised Goal-space Learning (UGL).

#### 2.2.3. IMGEPs With Learned Goal Spaces

Once a goal space has been learned using Algorithm 2, it can be combined with a goal exploration process. The procedure is summarized in Algorithm 3. When using a learned goal space, the agent samples goals in the learned goal space using the goal sampling distribution (line 10 of Algorithm 3). Similarly, the meta-policy learns to reach goals in the embedded space (line 14 of Algorithm 3). The overall approach combining IMGEPs with learned goal spaces is summarized in Figure 1.

**Algorithm 3:**
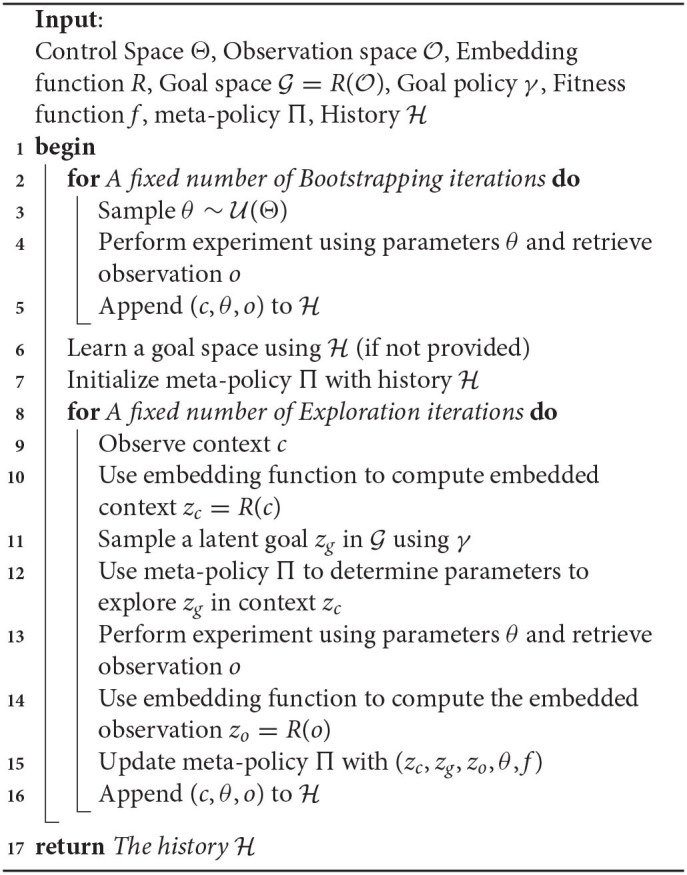
Random Goal Exploration with Unsupervised Goal space Learning (RGE-UGL).

## 3. Experiments

We carried out experiments both in a simulated environment and in a real-world robotic environment to address the following questions:

- How does an IMGEP using a learned goal space compare in terms of performance to an IMGEP using an engineered goal space?
- To what extent can the ideas developed in simulated environments be applied on a real-world setup.
- Does the dataset used to train the representation algorithm need to contain examples of all possible outcomes to learn a goal space that provides good performances during exploration.
- Another related question is whether the representation can be learned during exploration, as examples of outcomes are collected.

### 3.1. Environments

#### 3.1.1. Simulated Environment

**Figure 3:**
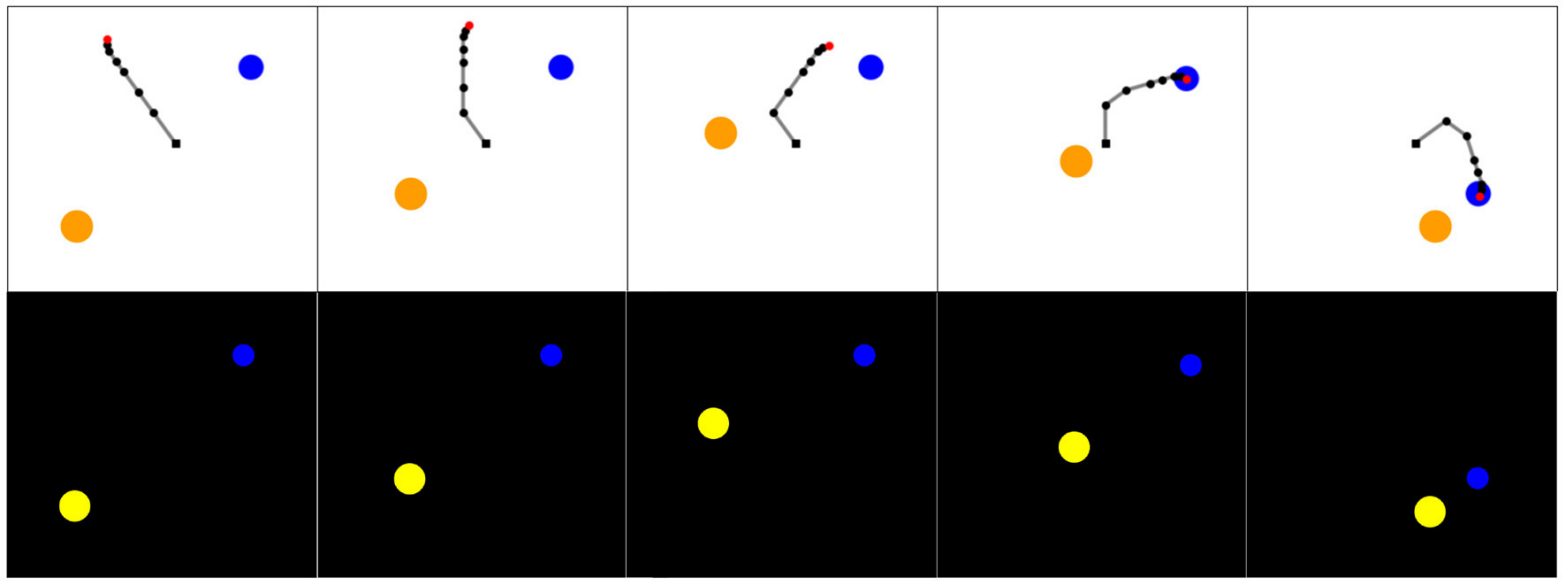
**(Top)** Visualization of a roll-out of experiment in the *Arm-2-Balls* environment. The blue ball can be grasped and moved, while the orange one is a distractor that cannot be handled and follows a random walk. The robotic arm is a 7-joint robotic arm controlled by DMPs. **(Bottom)** Observations as processed by the learning agents. The agent perceives the environment as 64 × 64 RGB images.

Simulated experiments were performed on the **Arm-2-Balls** environment. In this environment, a rotating 7-joint robotic arm evolves in a space containing two balls of different sizes, as represented in Figure 3. One ball can be grasped and moved around in the scene by the robotic arm. The other ball acts as a distractor: it cannot be grasped nor moved by the robotic arm but follows a random walk. The agent perceives the scene as a 64 × 64 RGB images. One episode in this environment corresponds to 50 timesteps. The action space is continuous and 56 dimensional, with actions corresponding to the parameters of the DMPs used during the roll-out (details on the motor control are given below). The DMPs used in one episode determines the position of each joint of the robotic arm at each timestep of the episode.

#### 3.1.2. Robotic Environment

**Figure 4:**
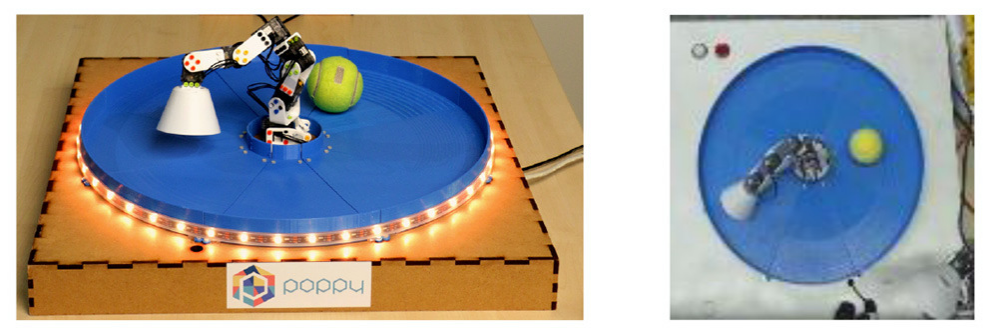
**(Left)** The robotic setup. It consists of a 6-joint robotic arm and a ball that is constrained to move in an arena. **(Right)** Example of an observation as perceived by the agent on the experimental platform. The observation is a 64 × 64 pixel RGB image.

The robotic setup is similar in spirit to the environment considered in the simulated experiments. The environment is composed of a 6-joint robotic arm [the open-source ErgoJr robot (Noirpoudre et al., 2017) designed using the Poppy platform (Lapeyre et al., 2014)] that evolves in an arena. In this arena a (tennis) ball can be moved around. Due to the geometry of the arena, the ball is more or less constrained to evolve on a circle. A picture of the environment is represented in Figure 4. The agent perceives the scene as a 64 × 64 pixels image. One episode in this environment corresponds to 40 timesteps. The action space is continuous and 49 dimensional, with actions corresponding to the parameters of the DMPs used during the roll-out (see next subsection for details). The DMPs determines the joints positions at each timestep of the episode.

#### 3.1.3. Motor Control

In both environments the motion of the arm is controlled by Dynamical Movement Primitives (DMPs) (Ijspeert et al., 2013). DMPs have been shown to allow fitting a wide range of behaviors using few parameters (Nakanishi et al., 2004; Pastor et al., 2013). In this work we take a reverse approach and use random parameters for the DMPs to produce a diversity of trajectories. Using the framework of DMPs to generate trajectories for the joints of the robotic arm allows for creating a wide range of diverse trajectories with few parameters (Forestier and Oudeyer, 2016; Forestier et al., 2017). Similar to how parameter space noise makes a reinforcement learning agent's exploration consistent across different timesteps (Plappert et al., 2018), DMPs help the agent perform consistent exploration unlike using random actions.

In both environments, each of the arm's degrees-of-freedom (DOF) is controlled by a DMP starting at the rest position of the joint. Each DMP is parameterized by one weight on each of the basic functions and one weight specifying the end position of the movement. In order to generate a new trajectory, a set of weights *w*_*i*_ and a goal *g* is sampled for each degree-of-freedom. The weights are bounded in the interval [−1, 1] and allow each joint to fairly cover the interval [−1, 1] during the movement. Each DMP outputs a series of positions that represents a sampling of the trajectory of one joint during the movement. Details on the DMPs framework are given in the Supplementary Material.

Actions in both environments are the parameters of the DMPs used in the current episode. Both environments use DMPs with 7 basis functions. As a result, the action space is 56 dimensional in the simulated environment and 49 dimensional in the real-world robotic experiment.

#### 3.1.4. Goal Space Learning

For the representation learning phase, we considered different strategies. In the first strategy, we considered that the agent has access to a database of examples of the possible set of outcomes. From this database the agent learns a representation that is then used as a goal space for the exploration phase. In both cases, the representation used as a goal space was learned using a VAE. In the simulated case, images were generated by uniformly sampling ball positions in [−1, 1]^4^, whereas in the real-world experiment the representation was trained using a database of examples^2^. Goal exploration experiments using this strategy for goal space learning are denoted with the suffix **VAE**.

One could argue that using this goal space learning strategy introduces knowledge on the set of possible outcomes that can be obtained by the agent. In order to test how this impacts the performances of the exploration algorithms we also experimented using a representation learned using only the samples collected during the initial iterations of random motor exploration. Experiments using this strategy to learn the goal space used for exploration are denoted with the suffix **Online**.

### 3.2. Exploration Strategies and Baselines

In this paper we consider exploration algorithms using a **Random Goal Exploration (RGE)** strategy with learned goal spaces. As described above, the goal space is learned either from a previously collected database (**RGE-VAE**) or from examples collected during exploration (**RGE-Online**).

These exploration algorithms are compared to two baselines:

- **Random Parameter Exploration (RPE)** In this case, the exploration is performed by uniformly sampling parameters θ. It corresponds to sampling a point in [−1, 1]^56^ and [−1, 1]^49^ for the simulated environment and robotic environment, respectively. This strategy does not leverage information collected during previous rollouts to choose the current parameters. Since DMPs were designed to enable the production of a diversity of arm trajectories with only a few parameters, this lower bound is already a solid baseline that performs better than applying random joint torques at each time-step of the episode.
- **Random Goal Exploration with Engineered Features Representation (RGE-EFR)** It is an IMGEP in which the goal space is handcrafted and corresponds (as closely as possible) to the true degrees of freedom of the environment. It corresponds to the position of the ball and of the distractor in the simulated environment and to the position of the ball and of the end effector in the robotic experiment. Since essentially all the information is available to the agent under a highly semantic form, it is expected to give an upper bound on the performances of the exploration algorithms.

## 4. Results

For the simulated experiment we performed 20 trials of 10,000 episodes each, for both the **RPE** and **RGE** exploration algorithms with both learned and engineered goal spaces. For the robotic experiment, we performed between 8 and 14 trials of 5,000 episodes each, for the **RPE** and **RGE** exploration algorithms with both engineered and learned goal spaces (learned on a fixed dataset or learned on examples collected during exploration)^3^. One episode is defined as one experimentation/roll-out of a parameter θ.

In both cases the end position of the robotic arm can be efficiently explored by performing random motor commands. Consequently, we define the performance of each exploration algorithm as the number of different ball positions reached during the experiment. It is measured according to the number of cells reached by the ball in a discretized grid of 900 cells (30 cells for each dimension). In the simulated experiment the distractor is not accounted for in the exploration evaluation since it cannot be controlled. For both experiments, not all cells can be reached given that the arm is rotating and is of limited length. In the simulated case, the maximum ratio between the number of reached cells and all the cells is approximately π/4 ≈ 0.8.

### 4.1. Simulated Environment

**Figure 5:**
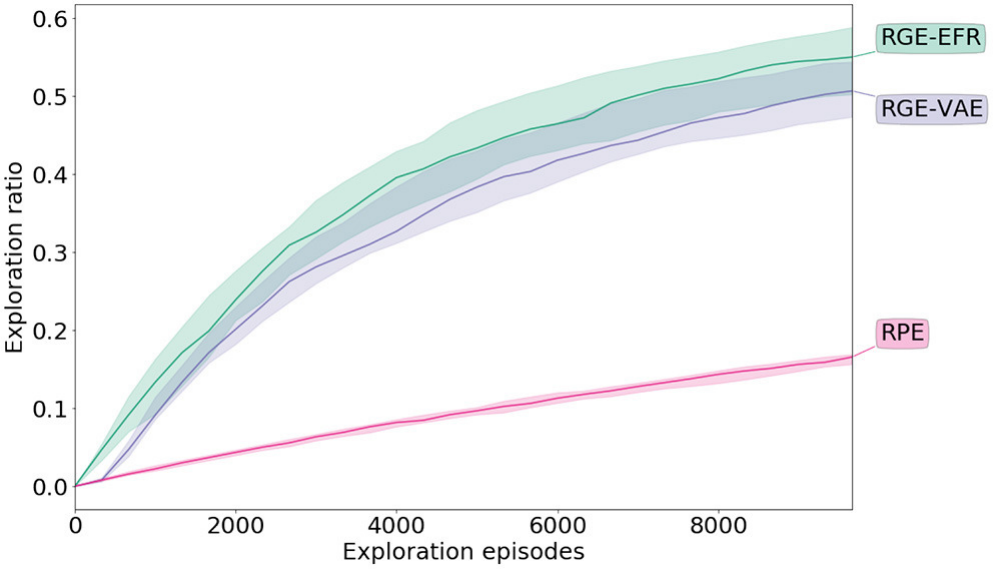
Exploration performance during exploration in the **Arm-2-Balls** experiment. Exploration is measured by discretizing the outcome space (possible end positions of the ball that can be controlled) and measuring the ratio of reached cells with respect to all the cells. The mean along with the inter-quartiles are depicted.

Figure 5 shows the evolution of the ratio of the number of cells visited with respect to all the cells through exploration. Both goal exploration algorithms perform much better than the naive **RPE**, both in term of speed of exploration and final performance (Welch's *t*-test *p* < 0.01). After the initial phase of random parameterization iterations, the goal exploration algorithms discover new outcomes faster than the **RPE** algorithm. In particular, the goal exploration algorithm using a learned representation as a goal space (**RGE-VAE**) performs similarly to the goal exploration algorithm using an engineered goal space (**RGE-EFR**) (*p* = 0.04 for the Welch's *t*-test). More examples of exploration curves together with exploration scatters are given in the Supplementary Material.

### 4.2. Robotic Environment

**Figure 6:**
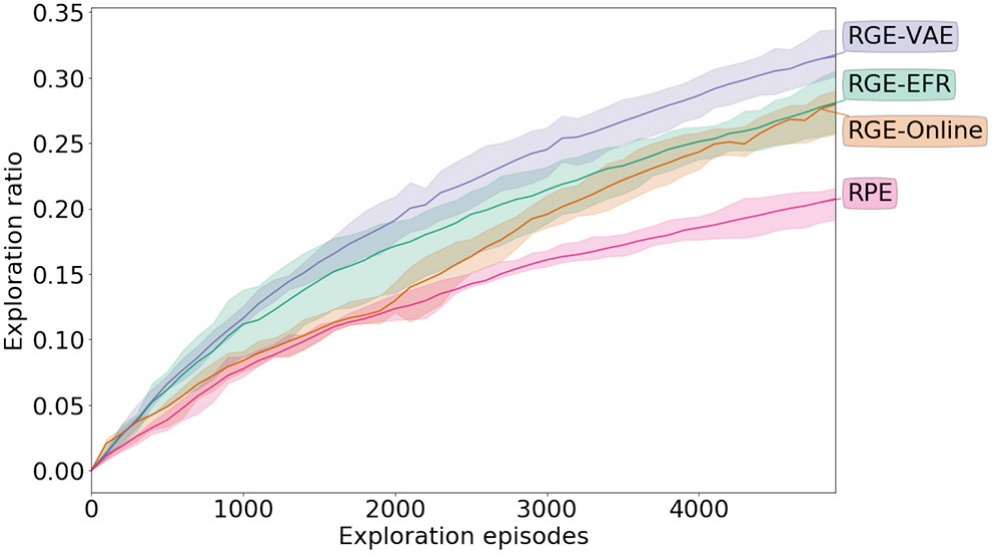
Exploration performance during exploration in the **real-world** robotic experiment. Exploration is measured by discretizing the outcome space (possible end positions of the ball) and measuring the ratio of reached cells with respect to all the cells. The mean along with the inter-quartiles are depicted.

The exploration performances are reported in Figure 6. From the plot, it is clear that IMGEPs with both learned and engineered goal spaces perform better than the **RPE** strategy (Welch's *t*-test *p* < 0.01). In the case when the representation is learned before exploration (**RGE-VAE**) the performances are at least as good as exploration using the engineered representation. When the goal space is learned using the online strategy, there is an initial phase where the exploration performances are the same as **RPE**. However, after this initial collection phase, when the exploration strategy is switched from random parameter exploration to goal exploration using the learned goal space (at 2, 000 exploration episodes) there is a clear change in the slope of the curve in favor of the goal exploration algorithm.

For the robot experiments, the differences in performances between IMGEPs and random parameter exploration are less pronounced than in past simulated experiments. We hypothesize that this is due to the ball being too simple to move around. Thus, the random parameter exploration, which leverages DMPs to produce diverse arm trajectories, achieves decent exploration results. Also, the motors of the robotic arm are far from being as precise as in the simulation, which makes it harder to learn a good inverse model for the policy and to output parameters that will move the ball.

**Figure 7:**
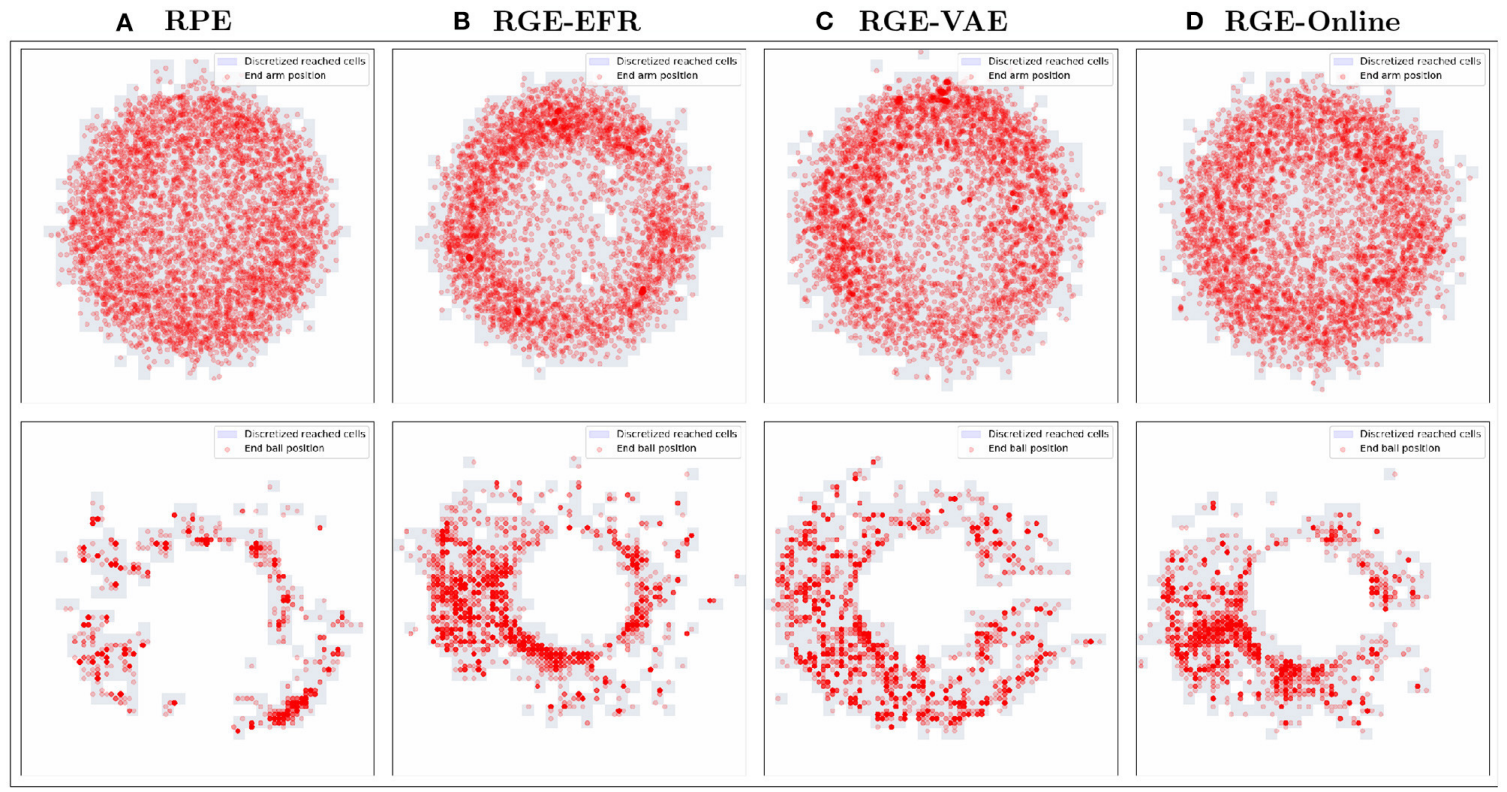
Scatter plots of the end of arm (top) and ball (bottom) positions visited during exploration for the **RPE (A)**, **RGE-EFR (B)**, **RGE-VAE (C)** and **RGE-Online (D)** exploration algorithms.

Scatter plots of the outcomes obtained during exploration are represented in Figure 7. Although the exploration of the outcome space of the arm is similar for all algorithms there is a qualitative difference in the outcomes obtained in the outcome space of the ball between **RPE** and all goal exploration algorithms.

## 5. Related Work

**Exploration**. Given the importance of exploration to solve complex problems and the limitations of current algorithms, many approaches have been developed to help agents explore efficiently.

One way to improve an agent's performances is to provide the agent with demonstrations that (partially) solve the task and to encourage the agent to reproduce those behaviors (Argall et al., 2009; Hester et al., 2018). More generally, one way to improve the agent's performance is to provide the agent with rich behaviors that produce a wide diversity of behaviors (Lynch et al., 2020). However, those behaviors must first be collected by a human, which is often expensive and time consuming. This might not even be feasible, for example when the association between action and outcome is unknown to the supervisor (Reinke et al., 2020). Also, these approaches require to have access to the motor commands used in the demonstration so that the agent can learn to imitate them. However, from a developmental perspective, these “motor programs” are not accessible to the children. Yet, infants are able to leverage observations gathered from their environment to efficiently learn new skills. From a developmental perspective it is thus also important to understand how partial demonstrations or examples can be used to bootstrap the learning of a new skill.

Another popular approach has been to augment reinforcement learning agents with intrinsic rewards to improve exploration. These intrinsic rewards generally value states or actions in terms of novelty, information gain, or prediction errors (Barto, 2013; Bellemare et al., 2016; Hester and Stone, 2017; Machado et al., 2017; Pathak et al., 2017). These rewards are designed to help the agent explore autonomously in the absence of an external signal by simulating some form of curiosity (Oudeyer, 2018), similar to the spontaneous exploration displayed by human children (Berlyne, 1966). Even when the true reward is suppressed, the signal provided by the intrinsic reward has been shown to enable agents to achieve good performances in a number of game environments (Burda et al., 2019).

Often, trying to directly optimize a given objective is not efficient because there may be deceptive rewards leading to bad local optima. Based on this observation, algorithms that find efficient solutions by purely valuing the novelty or diversity of discovered behaviors have been developed (Lehman and Stanley, 2011a; Eysenbach et al., 2019). Variants that combine a measure of novelty with a measure of fitness have also been developed (Lehman and Stanley, 2011b). These approaches share many similarities with intrinsically motivated goal exploration processes. In particular, IMGEPs with random goal sampling functions and implemented using nearest neighbors are equivalent to novelty search in the goal space (Portelas et al., 2020). More generally, intrinsically motivated goal exploration processes do not value novelty directly. Rather, they discover which goals are feasible or not, and leverage reachable goals to explore efficiently.

During exploration, it often happens that an agent fails to reach a given goal but still discovers another behavior. In order to learn efficiently, it is important that the agent be able to leverage those “failed” experiences. Intrinsically motivated goal exploration processes are able to leverage such experiences by storing an archive of policy parameters and their corresponding outcomes, enabling them to learn and discover new behaviors efficiently. Related approaches were also experimented in the context of Deep Reinforcement Learning, such as Hindsight Experience Replay (Andrychowicz et al., 2017) and Reverse Curriculum Learning (Florensa et al., 2017), and within the Power Play framework (Schmidhuber, 2013). Approaches such as Hindsight Experience Replay are often much more memory efficient and can be combined with IMGEPs when the number of examples becomes large (Colas et al., 2019).

**Representation learning for goal policies**. Other works have also considered how representation learning algorithms can be combined with policy learning algorithms.

Recently, Péré et al. (2018) showed that it is possible to use representations learned using a wide range of representation learning algorithms as goal spaces for IMGEPs, achieving a first step toward agents capable of autonomously learning a goal space in unknown environments. Specifically, a representation learning algorithm was used to learn a compact representation of the environment. This latent representation was then used as a goal space by the learning agent. Experiments performed in Péré et al. (2018) showed that goal exploration algorithms using learned goal spaces have similar exploration performances as goal exploration algorithms using handcrafted goal spaces. This idea was later extended to modular goal exploration processes (Laversanne-Finot et al., 2018). Such exploration algorithms are designed to efficiently explore environments that contain multiple objects or distractors (e.g., objects that cannot be controlled).

Experiments by Péré et al. (2018) and Laversanne-Finot et al. (2018), were performed in simulated environments, and a pre-collected database of possible outcomes was used to train the representation before exploration. This process limited the applicability of the approach as one needed to collect a representative database of possible examples which is hardly possible, for instance, when one does not know the set of possible outcomes (Reinke et al., 2020). Our work focuses on population based approaches and shows that the idea developed in Péré et al. (2018) can be applied to real world robotic experiments using only data collected by the learning agent. Similar ideas were experimented in parallel by Reinke et al. (2020), in the context of cellular automata. Reinke et al. (2020) showed that goal spaces learned online, using only data collected during exploration allows goal exploration algorithms to discover a wide range of behaviors in the continuous game of life Lenia.

Other works have also leveraged the power of deep representation algorithms in the context of population-based divergent search algorithms (Cully, 2019; Paolo et al., 2020). In these works, a low dimensional representation of the environment is learned during exploration and serves as behavioral descriptors for Novelty-Search or Quality-Diversity exploration algorithms.

**Reinforcement Learning**. Similar ideas have also been considered in the context of deep reinforcement learning. For example, Nair et al. (2018) extended the work of Péré et al. (2018) to deep reinforcement learning policies. Specifically, they learn a goal-conditioned policy that operates on a latent representation of the environment using a Variational Autoencoder (VAE). Results show that this method enables real world robots to perform a simple pushing task. This work was later extended by showing that it is possible to improve the exploration power of the algorithm by re-weighting the goal sampling probability, allowing robots to learn more complex tasks such as door opening (Pong et al., 2020).

More recently, learning goal conditioned policies have been the focus of a lot of work. For example, Warde-Farley et al. (2019) learn a goal policy by maximizing the mutual information between the goal state and the achieved state. Other works first learn a distance between states as the number of actions required to reach one state from another. This distance function is then used as a reward signal during the training of a goal conditioned policy (Hartikainen et al., 2019; Venkattaramanujam et al., 2019). While learning goal conditioned policies is not the subject of this work, IMGEPs can also be implemented using goal conditioned policies (Colas et al., 2019; Kovač et al., 2020). Population based IMGEPs are often faster for bootstrapping exploration and less computationally intensive but are often less efficient at generalizing to novel goals or exploring complex environments than IMGEPs using goal conditioned policies.

## 6. Discussion and Conclusion

In many cases, engineering a goal space is not a simple task and one has to choose from different possibilities, which will possibly not give the same performances. As such, learning the goal space is an appealing alternative. In this paper we have shown how learned representations can be used as goal spaces for exploration algorithms. Our results obtained both in a simulated experiment and in a real-world experiment, show that using a representation as a goal space provides better exploration performances than a random exploration using Dynamical Movement Primitives, controllers that are designed to produce diverse arm trajectories.

Another advantage of using representations as goal spaces is that it removes the need to extract high level features of the environment, such as the positions of objects, which are often needed to design a goal space. Extracting such high-level features requires *ad-hoc* algorithms for each environment. Such algorithms are also prone to errors. For example, in the case of the **RGE-EFR** algorithm used here, the position of the ball is extracted using a handcrafted algorithm. It may happen that the extraction algorithm fails (e.g., when the ball is hidden by the robotic arm). In that case it may report wrong state values. Such problems make learning an inverse model harder, which in turn, reduces the exploration performances. On the other hand, learned representations are designed to be robust to small perturbations and are often capable of small generalization and will thus on average report more meaningful values, even on perturbed images and may help learning an inverse model even if the conditions change (e.g., luminosity in the room).

Finally, as mentioned in the introduction, it is possible to imagine more involved goal selection schemes [see Supplementary Material for a short description of the results described in Laversanne-Finot et al. (2018)] when the representation is disentangled. These goal selection schemes leverage the disentanglement of the representation to provide better exploration performances. We tested these ideas in the real-world robotic experiment and did not find any advantages in using those goal selection schemes. This is not surprising since there are no distractors in this experiment and the environment does not contain many objects with big differences in learnability. However, modular goal exploration processes are specifically designed to handle distractors or help when there are multiple objects which have different learnability profiles. Consequently, designing a real-world experiment with distractors or multiple objects with big difference in learnability, in order to test modular goal exploration processes with learned goal spaces, would be of great interest for future work. In this work we simply used the VAE prior to sample goals. One can envision making the goal selection probability distribution bias, such that it favors goals that were rarely seen in order to improve the exploration performances (Baranes and Oudeyer, 2013). Similar ideas were experimented in the context of deep reinforcement learning (Florensa et al., 2018; Pong et al., 2020).

An important aspect of this work was to be able to perform experiments on a real-world robotic experiment with limited computing resources. In this regard, population based IMGEPs are particularly suited to this task since they require little computing resources. In this work we used a population-based method to learn the goal policy, with a simple nearest neighbor algorithm. Such an approach is very efficient in terms of sample efficiency for discovering diverse skills in complex bodies and environments and allows the agent to leverage past experiences immediately (Forestier et al., 2017), without resorting to training tricks (Andrychowicz et al., 2017). However, this approach is often limited in its capacity to generalize to new contexts.

Using deep reinforcement learning algorithms allows for learning policies that generalize better in new contexts but requires a much higher computational and data cost. In future work it would be interesting to deploy IMGEPs using a state-of-the-art deep reinforcement learning algorithm (Colas et al., 2019; Kovač et al., 2020) in real world robots. One can also envision schemes that combine both approaches: first collect data using a fast exploration algorithm using a population-based method and then use the collected data to learn policies using a deep reinforcement learning algorithm (Colas et al., 2018). The computing resources of the robotic platform also limited the training of the representation in the online experiment: the representation was trained using the first thousand examples but was not updated further on. In future work it would be interesting to continuously train the representation during exploration as experimented with in other works in simulated environments (Cully, 2019; Paolo et al., 2020; Reinke et al., 2020).

## Data Availability Statement

The datasets presented in this study can be found in online repositories. The names of the repository/repositories and accession number(s) can be found in the article/Supplementary Material.

## Author Contributions

AL-F, AP, and P-YO designed the algorithms and experimental protocols. AP and AL-F carried out the simulation experiments. AL-F carried out the robot experiments with support from AP. AL-F wrote the manuscript with support from AP and P-YO. P-YO supervised the project. All authors contributed to the article and approved the submitted version.

## Conflict of Interest

The authors declare that the research was conducted in the absence of any commercial or financial relationships that could be construed as a potential conflict of interest.

## Footnotes

^1^The code used in the experiments is available at https://github.com/flowersteam/Curiosity_Driven_Goal_Exploration for the simulated experiments and https://github.com/flowersteam/APEX/tree/poppimage for the real-world robotic experiment.

^2^Details on the VAE architecture, training procedure and data collection are given in the Supplementary Material.

^3^The duration of a single experiment is around 12h.
