# Supplementary material for: Intrinsically Motivated Exploration of Learned Goal Spaces
Source: Front Neurorobot. 2021 Jan 12;14:555271. doi: 10.3389/fnbot.2020.555271 (PMC7835425; doi:10.3389/fnbot.2020.555271)
Supplement: Supplementary file 1 [file Data_Sheet_1.PDF]

Supplementary Material

1 APPENDICES

1.1 Modular Goal Exploration Processes

In this section we summarize some of the results presented in Laversanne-Finot et al. (2018) on modular goal exploration processes with learned goal spaces.

1.1.1 IMGEPs with modular goal spaces

1.1.1.1 General idea and algorithmic architecture

As mentioned in the main text, when the environment is more complex and in particular when it contains distractors (objects that cannot be controlled), it is possible to design more efficient exploration algorithms. Modular goal exploration algorithms are designed to allow the agent to separate the exploration of different objects. For example, in an environment containing a robotic arm and a ball, the agent could decide to set for himself either goals for the ball or for its arm. Simply choosing different types of goals at random often already leads to substantial progress over random goals over the whole environment since such goals are often impossible to realize (e.g. moving the ball without moving the arm) (Forestier and Oudeyer, 2016). An even better strategy is to select goals according to some measure of interest such as the learning progress. The general idea is that some goals are harder (if not impossible) to reach than others. By monitoring its ability in fulfilling different kinds of goals the agent will be able to discover autonomously the difficulty of

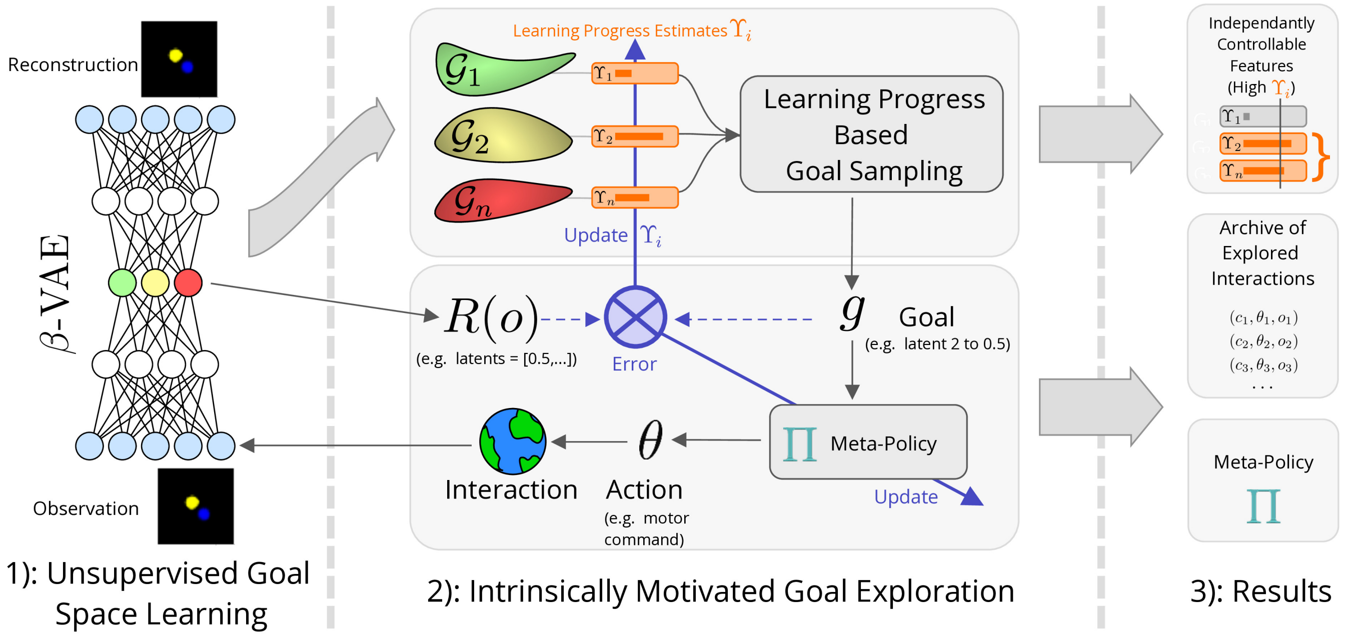

Figure S1. Intrinsically Motivated Goal Exploration Processes with Modular Unsupervided Goal Space Learning (IMGEP-MUGL).

each type of goals and focus its exploration on goals which are neither too easy nor too hard. Using this strategy the agents thus autonomously design a curriculum. In the case of environments with engineered goal spaces this strategy has been shown to enable robots (Forestier and Oudeyer, 2016; Forestier et al., 2017) to automatically generate a learning curriculum and ultimately to discover a wide range of behaviors. See Algorithm 4 for a sketch of the corresponding algorithmic architecture.

Algorithm 4: Modular Goal Exploration

begin
  while *Exploration not finished* **do**
    Observe context c
    Sample a module i using the module sampling distribution
    Sample a goal g_i inside module i
    Determine parameters to reach goal g_i given context c using Meta-Policy
    Perform experiment and collect observation o
    Update the module sampling distribution in order to track the learning progress

1.1.1.2 Goal space learning for modular goal exploration processes

When the goal space is engineered, the different modules can be readily defined when designing the goal space. However, in the case of learned goal spaces there is no straightforward solution. The strategy proposed in Laversanne-Finot et al. (2018) is to form *modules* by grouping some of the latent variables together. The goals of one module are then to reach observations for which the latent variables corresponding to this module have specific values. The procedure for learning the different goal modules is outlined in Algorithmic Architecture 5.

Algorithm 5: Modular Unsupervised Goal-space Learning (MUGL)

Input:
Representation learning algorithm $\mathfrak{R}$ (e.g. VAE, βVAE), Kernel Density Estimator algorithm $\mathfrak{E}$
begin
  for *A fixed number of Observation iterations n_r* **do**
    Observe external agent produce observation o_i
    Append this sample to database $\mathcal{D}_o = \{o_i\}_{i=0,\ldots,n_r}$
  Learn an embedding function $R : \mathcal{O} \mapsto \mathbb{R}^{n_d}$ using algorithm $\mathfrak{R}$ on data $\mathcal{D}_o$
  Generate an ensemble of projection operators $\{P_k\}$
  Estimate γ_k from $\{P_k R(o_i)\}_{i=0,\ldots,n_r}$ using algorithm $\mathfrak{E}$
  Set the fitness functions to be $f_k(o, g) = -||P_k R(o) - g||$
return *An embedding function R and goal modules $\{P_k, \gamma_k, f_k\}$.*

1.1.1.3 Active module sampling based on the learning progress

In a modular architecture the goal sampling policy reads:

$$\gamma(g) = \gamma(g|i)p(i), \tag{S1}$$

where $p(i)$ is the probability to sample the $\mathcal{G}_i$ module, and $\gamma(g|i)$ is the probability to sample the goal g given that the module i was selected. The strength of the modular architecture is that modules can be selected using a curiosity-driven *active* module sampling scheme. In this scheme, $\gamma(g|i)$ is fixed, and p is

updated during exploration. The general idea is to bias the module sampling probability in order to favor sampling goals in modules for which the agent is making progress, allowing the agent to concentrate on modules that produce goals which can be achieved while ignoring goals that cannot. This is achieved by monitoring the learning progress of each modules. In the next paragraph we detail this mechanism on one interest measure known as the competence progress.

In our case, the probability $\gamma(i)$ of sampling a goal from module i is given by:

$$p(i) := 0.9 \times \frac{\Upsilon_i(t)}{\sum_{k=1}^{N} \Upsilon_k(t)} + 0.1 \times \frac{1}{N}, \tag{S2}$$

where $\Upsilon_i(t)$ is the competence progress. The second term of Equation (S2) ensures that the agent explore using all the modules during exploration. Intuitively, the *competence progress* is an estimation of the average *improvement* of the precision of the meta-policy for fulfilling goals corresponding to the i-th module (see (Baranes and Oudeyer, 2013)). Sampling modules according to Equation (S2) ensures that the agent concentrates on goals for which he is making progress.

Let o_t and g_t be respectively the outcome and goal for the exploration step t. The progress δ_t^i in module i at step t is estimated as follows:

$$\delta_t^i = C_i(o', g_t) - C_i(o_t, g_t), \tag{S3}$$

where o' is the observation associated to the experiment in $\mathcal{H}$ for which the goal g' is the closest to g_t. The interest of a module is designed to track the progress. In practice, the interest of each module is updated according to:

$$\Upsilon_i(t) = \frac{n-1}{n}\Upsilon_i(t-1) + \frac{1}{n}\delta_t^i, \tag{S4}$$

where $n = 1000$ is a decay rate ensuring that the interest eventually decay to zero if no progress is made (see Forestier and Oudeyer (2016) for more details).

1.1.1.4 Modular goal exploration with learned goal spaces

Algorithm 6 provides detailed pseudo-code for how we combined unsupervised modular goal space learning with goal exploration. The main differences between the modular goal exploration strategy described in Algorithm 6 and the goal exploration strategy described in the main text is that instead of sampling only a goal, the agent first samples a module and then a goal in the goal space of that module. Accordingly, the Meta-Policy takes the corresponding module as an input in order to compute the best parameters for this particular goal. In practice this can be simply achieved by learning a goal policy for each module and using the corresponding goal policy. However, when the number of goals becomes large, it is more efficient to only learn one policy for all the different modules so that some cross transfer learning can happen between different types of goals (Colas et al., 2019).

1.1.2 Results of Modular Goal Exploration with learned goal spaces

The active learning strategy detailed in the last section is efficient if modules correspond to different objects or properties of the environment. If the modules are created from an engineered representation, it is usually easy to fulfill this requirement. However the problem remains when the goal space is learned, using a representation learning algorithm. The intuition developed in (Laversanne-Finot et al., 2018) is

Algorithm 6: Curiosity Driven Modular Goal Exploration with Unsupervised Goal space Learning (MGE-UGL)

Input:
Control Space Θ, Observation space $\mathcal{O}$, Embedding function R, Goal space $\mathcal{G} = R(\mathcal{O})$,
Goal modules $\{(P_i, \gamma_i, C_i)\}$, Meta-Policy Π, History $\mathcal{H}$

begin
  for *A fixed number of Bootstrapping iterations* **do**
    Observe context c
    Sample $\theta \sim \mathcal{U}(-1, 1)$
    Perform experiment using parameters θ and retrieve observation o
    Append (c, θ, o) to $\mathcal{H}$
  Initialize Meta-Policy Π with history $\mathcal{H}$
  Initialize module sampling probability $p = \mathcal{U}(n_{mod})$
  for *A fixed number of Exploration iterations* **do**
    Observe context c
    Sample a module $i \sim p$
    Sample a latent goal z_{g_i} in $\mathcal{G}_i$ using γ_i
    Use Meta-Policy Π to determine the parameters θ to reach goal z_{g_i} given context c
    Perform experiment using parameters θ and retrieve observation o
    Use embedding function to compute the embedded observation $z_o = R(o)$
    Update Meta-Policy Π with (c, z_{g_i}, z_o)
    Update module sampling probability p to follow learning progress according to (S4)
    Append (c, θ, o) to $\mathcal{H}$
return *The history* $\mathcal{H}$

to use a disentangled representation as the goal space. If the representation of the world is disentangled, different latent variables encode for different degrees of freedom of the environment. In that case modules will correspond to distinct objects corresponding to the latent variables of this module. By monitoring its progress in controlling each of the latent variables the agent will discover that latent variables that encode for distractors cannot be controlled while latent variables encoding for other objects can be controlled. The agent will thus be able to focus its exploration on controllable latent variables, leading to better exploration performances.

The ideas of modular IMGEPs were tested in the *Arm-2-Balls* environment described in the main text of the paper. In this section we detail the experiments performed in(Laversanne-Finot et al., 2018) comparing the exploration performances of modular goal exploration algorithms using disentangled or entangled representations as a goal space.

1.1.2.1 Goal space learning

For the representation learning phase we used a Variational Auto-Encoder (VAE) for the entangled representation and a β-VAE for the disentangled representation. β-VAE are a variant of VAEs that have been argued to have better disentanglement properties (Higgins et al., 2016, 2017b,a). To train the representation, we generated a dataset of images for which the positions of the two balls were uniformly distributed over $[-1, 1]^4$. This dataset was then used to learn a representation using a VAE or a β-VAE. In order to test the impact of the disentanglement on the performances of the exploration algorithms, we used the same disentangled/entangled representation for all the instantiations of the exploration algorithms. This allowed us to study the effect of disentangled representations by eliminating the variance due to the inherent difficulty of learning such representations.

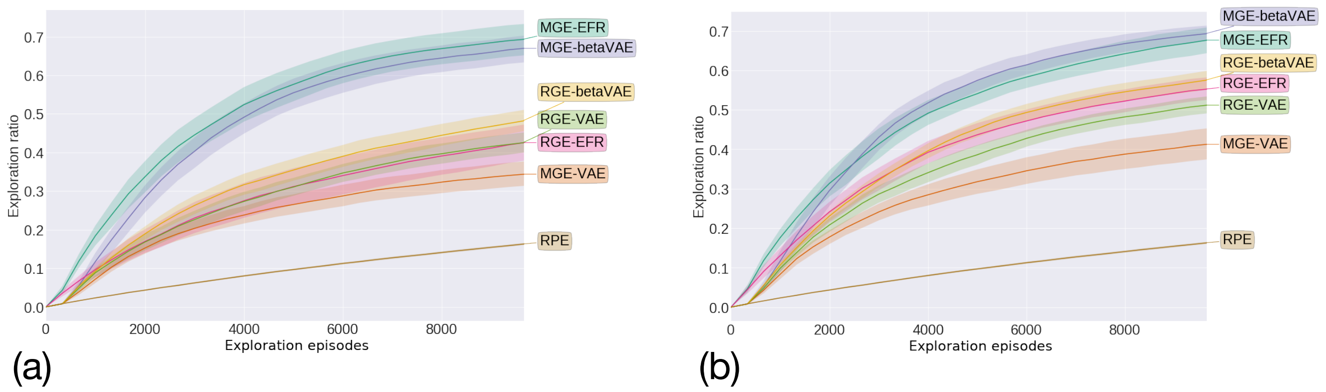

Figure S2. Exploration ratio during exploration for different exploration noises ((a) $\sigma = 0.05$ and (b) $\sigma = 0.1$.

1.1.2.2 Exploration performances

Figure S2, shows the evolution of the ratio of the number of cells visited with respect to all the cells through exploration for various exploration noises. When the representation used as a goal space is disentangled (βVAE), the performances obtained by modular goal exploration algorithms (**MGE-EFR** and **MGE-βVAE**) are well above regular goal exploration exploration algorithms (**RGE-VAE** and **RGE-EFR**). On the other hand, when the representation is entangled, the modular goal exploration algorithm (**MGE-VAE**) has poorer performances than its random goal exploration counterpart (**RGE-VAE**).

Examples of exploration curves using modular goal exploration algorithms are represented on Figure S3. Visual inspection shows that random parameterization exploration fails to produce a wide variety of outcomes and that on average goal exploration algorithms perform much better. However, although random goal exploration algorithms perform much better than the random parameterization algorithm, they tend to produce observations that are cluttered in a small region of the space. On the other hand, modular goal exploration algorithms with disentangled representation produce observations that are almost uniformly distributed over the reachable space.

1.1.2.3 Interest curves and independently controllable features

A byproduct of modular exploration algorithms is that by monitoring the progress in controlling certain features of the environment, the agent is capable of discriminating between features that can be controlled and those that cannot. This is particularly visible on the interest curves obtained during the training of the agent represented in figure S4: there is a clear difference in interest between modules where learning progress happens and modules for which the agent does not make any progress. A latent traversal study of the VAE shows that those modules correspond to latent variables that encode for the ball position. Extracting such information is valuable as it could be leveraged by another learning algorithm to speed up the training. Note that this phenomenon happens only when the representation is disentangled and latent variables encode for independent degrees of freedom of the environment.

1.2 Dynamical Movement Primitives (DMPs)

This section gives more details on Dynamical Movement Primitives. For more details, we refer the reader to Ijspeert et al. (2013).

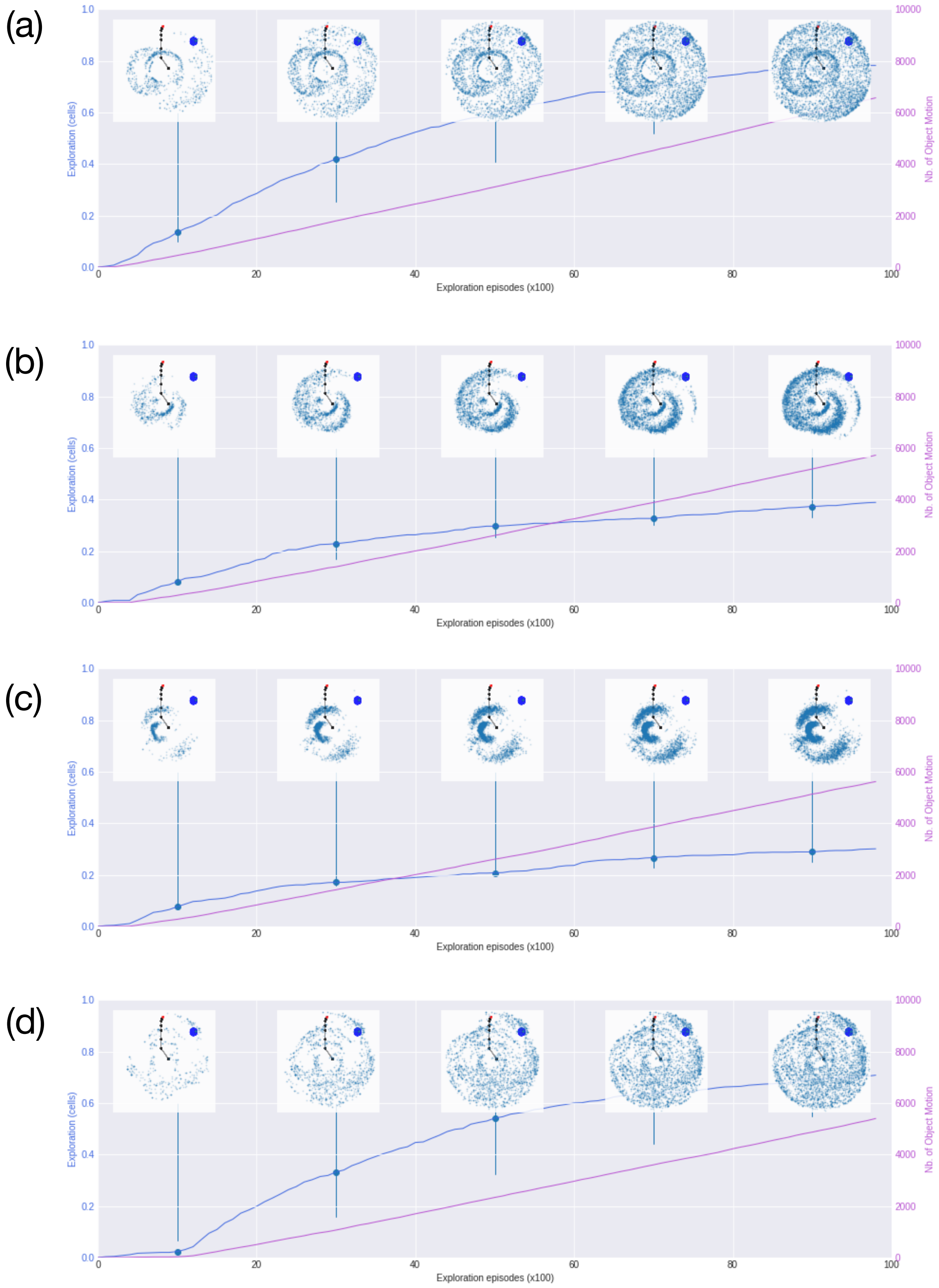

Figure S3. Examples of achieved observations together with the ratio of covered cells in the *Arm-2-Balls* environment for **MGE-EFR** (a), **RGE-βVAE** (b), **MGE-VAE** (c) and **MGE-βVAE** (d) exploration algorithms. The number of times the ball was effectively handled is also represented.

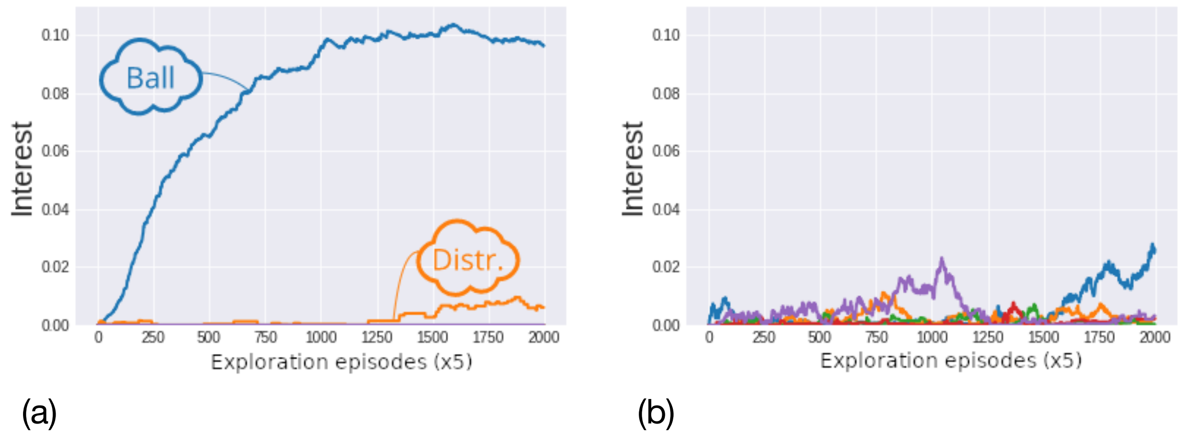

Figure S4. Interest evolution for each module through epochs. In the case of a disentangled representation (a) the algorithm shows interest only for the modules which correspond to latent variables encoding for the position of the ball (which is unknown by the agent, which does not distinguish between the ball and the distractor). For the entangled representation (b), the interest is similar for all modules.

To control the arm, one needs to generates (continuous) trajectories for the joints. The general idea of DMPs is to view each joint trajectory as the solution of a differential equation. Consider the following differential equation:

$$\tau \ddot{y} = \alpha(\beta(g - y) - \dot{y}) + f \tag{S5}$$

where τ, α and β are constants and f is a forcing term. If the forcing term $f = 0$, then this differential equation admits a unique attractor point $y = g$. For appropriate values of β and α ($\beta = \alpha/4$), the solution y of (S5) monotonically converges towards g. The general idea of DMPs is to tune f in order to generate more complex trajectories.

Let x be the solution of the following differential equation:

$$\tau \dot{x} = -\alpha x, \tag{S6}$$

where α is a constant. The variable x can be thought of as a phase variable. Starting from an arbitrary initial condition x_0, x decreases monotonically to 0. $x = x_0$ indicates the start of the time evolution while x close to zero indicates that the goal g has been reached. The forcing term f can be chosen to be:

$$f(x) = \frac{\sum_{i=1}^{N} \Psi_i(x) w_i}{\sum_{i=1}^{N} \Psi_i(x)} x(g - y_0), \tag{S7}$$

where Ψ_i are exponential basis functions, w_i are weights, y_0 is the initial position of the system. In (S7) the term $g - y_0$ serves as a scaling factor while the term x ensures that the forcing term vanishes when the goal g is reached. The whole system has a unique equilibrium point $(z, y, x) = (0, g, 0)$. From any initial condition y will evolves towards the goal g. However, adjusting the weights w_i allows to generate complex trajectories before reaching g.

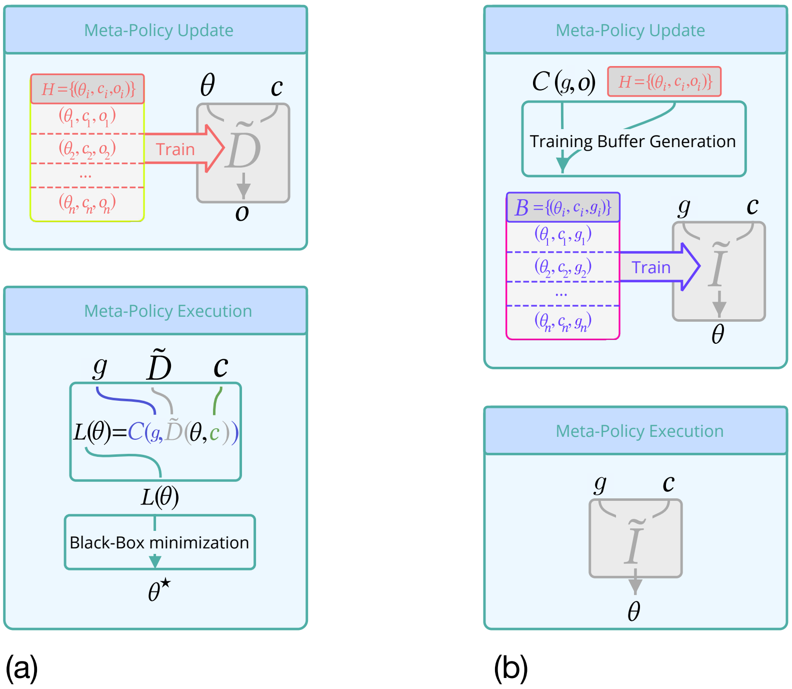

Figure S5. The two different approaches to construct a meta-policy mechanism: Direct-Model Meta-Policy (a) and Inverse-Model Meta-Policy (b).

1.3 Meta-Policy Mechanism

A Meta-Policy is a function that, given a context c and a goal g, outputs the parameters θ that are most likely to produce an observation o that reaches the goal g. In order to learn a Meta-Policy, it is necessary to quantify how much an observation o fulfills a goal g. This is done using a cost function $C : \mathcal{G} \times \mathcal{O} \mapsto \mathbb{R}$. The cost function can be seen as representing the fitness of the observation o regarding the task g.

The Meta-Policy can be constructed in two different ways which are depicted in Figure S5:

- **Direct-Model Meta-Policy:** In this case, an approximate phenomenon dynamic model $\tilde{D}$ is learned using a regressor (e.g. LWR). The model is then updated regularly by performing a training step with the newly acquired data. At execution time, for a given goal g, a loss function is defined over the parameterization space through $L(\theta) = C(g, \tilde{D}(\theta, c))$. A black-box optimization algorithm, such as L-BFGS, is then used to optimize this function and find the optimal set of parameters θ (see (Baranes and Oudeyer, 2013; Forestier and Oudeyer, 2016; Benureau and Oudeyer, 2016) for examples of such meta-policy implementations in the IMGEP framework).
- **Inverse-Model Meta-Policy:** Here, an inverse model $\tilde{I} : \mathcal{G} \times \mathcal{C} \mapsto \Theta$ is learned from the history $\mathcal{H}$ which contains all the previous experiments in the form of tuples $\{(c_i, \theta_i, o_i)\}$. To do so, every experiments observations o must be turned into a goal g. The inverse model can then be learned using usual regression techniques from the set $\{(g_i, c_i, \theta_i)\}$.

In our case, we took the approach of using an Inverse-Model based Meta-Policy. We draw the attention of the reader on the following implementation details: depending on the case, multiple observations, and consequently multiple parameters can optimally solve a task, while a combination of them cannot. This is known as the redundancy problem in robotics and special approaches must be used to handle it when learning inverse models, in particular within the IMGEP framework (Baranes and Oudeyer, 2013). This has

also been tackled under the terminology of multi-modality in (Pathak et al., 2018). To solve this problem, we used a κ-nn regressor with $\kappa = 1$.

Our particular implementation of the Meta-Policy is outlined in Algorithm 7. The Meta-Policy is instantiated with one database that stores the representations of the observations with the associated contexts and parameterizations. Given that the meta policy is implemented with a nearest neighbor regressor, training the meta policy simply amounts to updating all the databases.

Algorithm 7: Meta-Policy (simple implementation using a nearest-neighbor model)

Function `Initialize_Meta-Policy(`$\mathcal{H}$`)`:
- database $\leftarrow$ VoidDatabase
- **for** $(c, \theta, o) \in \mathcal{H}$ **do**
  - Add (c, θ, o) to database

Function `Update_Meta-Policy(`c, θ, o`)`:
- Add $(c, \theta, R(o))$ to database

Function `Infer_parameterization(`c, g, σ`)`:
- $\theta \leftarrow$ NearestNeighbor(database, c, g)
- $\theta \leftarrow \theta + \mathcal{N}(0, \sigma)$
- **return** θ

1.4 Deep Representation Learning Algorithms

In this section we summarize the theoretical arguments behind Variational AutoEncoder (VAE) and βVAE.

1.4.1 Variational Auto-Encoders (VAEs)

Let $\mathbf{x} \in \mathcal{X}$ be a set of observations. If we assume that the observed data are realizations of a random variable, we can hypothesize that they are conditioned by a random vector of independent factors $\mathbf{z}$, i.e. that $p(\mathbf{x}, \mathbf{z}) = p(\mathbf{z})p_\theta(\mathbf{x}, \mathbf{z})$, where $p(\mathbf{z})$ is a *prior* distribution over z and $p_\theta(\mathbf{x}, \mathbf{z})$ is a *conditional distribution*. In this setting, given a i.i.d dataset $X = \{\mathbf{x}^1, \ldots, \mathbf{x}^N\}$, learning the model amounts to searching the parameters θ that maximizes the dataset likelihood:

$$\log \mathfrak{L}(\mathcal{D}) = \sum_{i=1}^{N} \log p_\theta(\mathbf{x}^i) \tag{S8}$$

However, in most cases, the marginal probability:

$$p_\theta(\mathbf{x}) = \int p(\mathbf{x}, \mathbf{z}) \mathrm{d}\mathbf{z} \tag{S9}$$

and the posterior probability:

$$p_\theta(\mathbf{z}|\mathbf{x}) = \frac{p(\mathbf{x}, \mathbf{z})}{p(\mathbf{z})} = \frac{p(\mathbf{x}, \mathbf{z})}{\int p(\mathbf{x}, \mathbf{z}) \mathrm{d}\mathbf{z}} \tag{S10}$$

are both computationally intractable, making the maximum likelihood estimation unfeasible. To overcome this problem, we can introduce an arbitrary distribution $q_\phi(\mathbf{z}|\mathbf{x})$ and remark that the following holds:

$$\log p_\theta(\mathbf{x}) = \mathcal{L}(\mathbf{x}; \theta, \phi) + \mathbb{D}_{KL}\left[q_\phi(\mathbf{z}|\mathbf{x}) \| p_\theta(\mathbf{z}|\mathbf{x})\right], \tag{S11}$$

where $\mathbb{D}_{KL}$ denotes the Kullback-Leibler (KL) divergence and

$$\mathcal{L}(\mathbf{x}; \theta, \phi) = \mathbb{E}_{\mathbf{z}\sim q_\phi(\mathbf{z}|\mathbf{x})}[\log p_\theta(\mathbf{x}|\mathbf{z})] - \mathbb{D}_{KL}[q_\phi(\mathbf{z}|\mathbf{x}) \| p(\mathbf{z})]. \tag{S12}$$

Since the KL divergence is non-negative, it follows from (S11) that:

$$\mathcal{L}(\mathbf{x}; \theta, \phi) \leq \log p_\theta(\mathbf{x}) - \mathbb{D}_{KL}[q_\phi(\mathbf{z}|\mathbf{x}) \| p_\theta(\mathbf{z}|\mathbf{x})] \tag{S13}$$

for any distribution q, hence the name of Evidence Lower Bound (ELBO). Consequently, maximizing the ELBO has the effect to maximize the log likelihood, while minimizing the KL-Divergence between the approximate $q_\phi(\mathbf{z}|\mathbf{x})$ distribution, and the true unknown posterior $p_\theta(\mathbf{z}|\mathbf{x})$. The approach taken by VAEs is to *learn* the parameters of both conditional distributions $p_\theta(\mathbf{x}|\mathbf{z})$ and $q_\phi(\mathbf{z}|\mathbf{x})$ as non-linear functions. This is done by maximizing the ELBO of the dataset:

$$\mathcal{L}(\theta, \phi) = \sum_{i=1}^{N} \mathcal{L}(\mathbf{x}^i; \theta, \phi) \tag{S14}$$

by jointly optimizing over the parameters θ and ϕ. When the prior $p(\mathbf{z})$ is an isotropic unit Gaussian distribution and the variational approximation $q_\phi(\mathbf{z}|\mathbf{x})$ follows a Multivariate Gaussian distribution with diagonal covariance, the KL divergence term can be computed in a closed form.

In essence, a VAE can be understood as an AutoEncoder with stochastic units ($q_\phi(\mathbf{z}|\mathbf{x})$ plays the role of an encoder while $p_\theta(\mathbf{x}|\mathbf{z})$ plays the role of the decoder), together with a regularization term given by the KL divergence between the approximation of the posterior and the prior. The existence of a prior over the latent variables gives the ability to use a VAE as a generative model, and latent variables sampled according to the prior $p(\mathbf{z})$ can be transformed by the decoder into samples.

1.4.2 Details of Neural Architectures and training

1.4.2.1 Model Architecture

In both experiments the encoder for the VAEs consisted of 4 convolutional layers, each with 32 channels, 4x4 kernels, and a stride of 2. This was followed by 2 fully connected layers, each of 256 units. The latent distribution consisted of one fully connected layer of 20 units parametrizing the mean and log standard deviation of 10 Gaussian random variables. The decoder architecture was the transpose of the encoder, with the output parametrizing Bernoulli distributions over the pixels. ReLu were used as activation functions. This architecture is based on the one proposed in (Higgins et al., 2016).

1.4.2.2 Training details

1.4.2.2.1 Simulated experiment

For the training of the representation the optimizer used was Adam (Kingma and Ba, 2015) with a learning rate of $5e^{-5}$ and batch size of 64. The overall training of the representation took 1M training iterations.

1.4.2.2.2 Real-world robotic experiment

For the **RGE (VAE)** experiement the representation was learned using a database of 17k examples of possible outcomes. The database was obtained by running random motor commands and then filtered to obtain a database of examples whose true state distribution is as close as possible to the uniform distribution. The network was trained using the Adam optimizer (Kingma and Ba, 2015) with a learning rate of $1e^{-5}$ and batch size of 64. The training procedure of the VAE took 26k iterations.

In the online experiment (**RGE (Online)**) 2000 examples were collected using a random policy. Those examples were then used to learn the VAE which was later used as a goal space. The network was trained using the Adam optimizer (Kingma and Ba, 2015) with a learning rate of $1e^{-5}$ and batch size of 32. The VAE was trained for 10k iterations.

Robotic experiments are performed using several robots and in order to speed up the learning procedure we used the same representation for all trials performed on a specific robot. We did not pick a particular representation and control experiments show that similar performances are obtained for other representations.

1.5 Statistical comparison of each exploration algorithms

Table S1 and Table S2 report the Welch's t-test p-values obtained when comparing each pair of exploration algorithms.

	**RPE**	**RGE-EFR**	**RGE-VAE**	**RGE-βVAE**	**MGE-EFR**	**MGE-VAE**	**MGE-βVAE**
**RPE**	1	5e-8	9e-12	2e-12	5e-13	2e-8	1e-13
**RGE-EFR**	5e-8	1	0.99	0.06	9e-9	0.011	2e-8
**RGE-VAE**	9e-12	0.99	1	8e-3	2e-10	6e-4	6e-11
**RGE-βVAE**	2e-12	6e-2	8e-3	1	2e-8	8e-7	2e-8
**MGE-EFR**	5e-13	9e-9	2e-10	2e-8	1	7e-13	0.4
**MGE-VAE**	2e-8	0.011	6e-4	8e-7	7e-13	1	2e-13
**MGE-βVAE**	1e-13	2e-8	6e-11	2e-8	0.4	2e-13	1

Table S1. Welch's t-test p-values for the simulated experiment.

	**RPE**	**RGE-EFR**	**RGE-VAE**	**RGE-Online**
**RPE**	1	7e-3	4e-9	2e-5
**RGE-EFR**	7e-3	1	0.1	0.9
**RGE-VAE**	4e-9	0.1	1	0.011
**RGE-Online**	2e-5	0.9	0.011	1

Table S2. Welch's t-test p-values for the robotic experiment.

1.6 Hyperparameters

Table S3 and Table S4 list the hyperparameters used for the simulated experiments.

Table S5 lists the hyperparameters used for the robotic experiments.

**Hyperparameter**	**RPE**	**RGE-EFR**	**RGE-VAE**	**RGE-βVAE**
Action dimension	56	56	56	56
Observation dimension	N/A	4	64x64x3	64x64x3
Bootstrapping iterations	10 000	400	400	400
Exploration noise	N/A	0.1	0.1	0.1
Exploration iterations	N/A	9 600	9 600	9 600
Observation iterations (n_r)	N/A	N/A	160 000	160 000
Context dimension	N/A	N/A	N/A	N/A
Goal space	N/A	Engineered (x, y positions of ball and distractor)	Learned (VAE)	Learned (βVAE)
Embedding dimension (n_d)	N/A	N/A	10	10
Number of modules	N/A	N/A	N/A	N/A
Goal space dimension	N/A	4	10	10
Interest decay rate (Eq. (S4))	N/A	N/A	N/A	N/A

Table S3. Hyper-parameters used for the simulated experiments.

**Hyperparameter**	**MGE-EFR**	**MGE-VAE**	**MGE-βVAE**
Action dimension	56	56	56
Observation dimension	4	64x64x3	64x64x3
Bootstrapping iterations	400	400	400
Exploration noise	0.1	0.1	0.1
Exploration iterations	9 600	9 600	9 600
Observation iterations (n_r)	N/A	160 000	160 000
Context dimension	N/A	N/A	N/A
Goal space	Engineered (x, y positions of ball and distractor)	Learned (VAE)	Learned (βVAE)
Embedding dimension (n_d)	N/A	10	10
Number of modules	2 or 4	5 or 10	5 or 10
Goal space dimension	2 or 1	2 or 1	2 or 1
Interest decay rate (Eq. (S4))	1000	1000	1000

Table S4. Hyper-parameters used for the simulated experiments.

**Hyperparameter**	**RPE**	**RGE-EFR**	**RGE-VAE**	**RGE-Online**
Action dimension	49	49	49	49
Observation dimension	N/A	4	64x64x3	64x64x3
Bootstrapping iterations	10 000	100	100	2 000
Exploration noise	N/A	0.5	0.5	0.5
Exploration iterations	N/A	9 900	9 900	8 000
Observation iterations (n_r)	N/A	N/A	26 415	0
Context dimension	N/A	2	10	10
Goal space	N/A	Engineered (x, y pos. of ball and x, y, z pos. of end-effector)	Learned (VAE)	Learned (βVAE)
Embedding dimension (n_d)	N/A	N/A	10	10
Number of modules	N/A	N/A	N/A	N/A
Goal space dimension	N/A	5	10	10

Table S5. Hyper-parameters used for the robotic experiments.

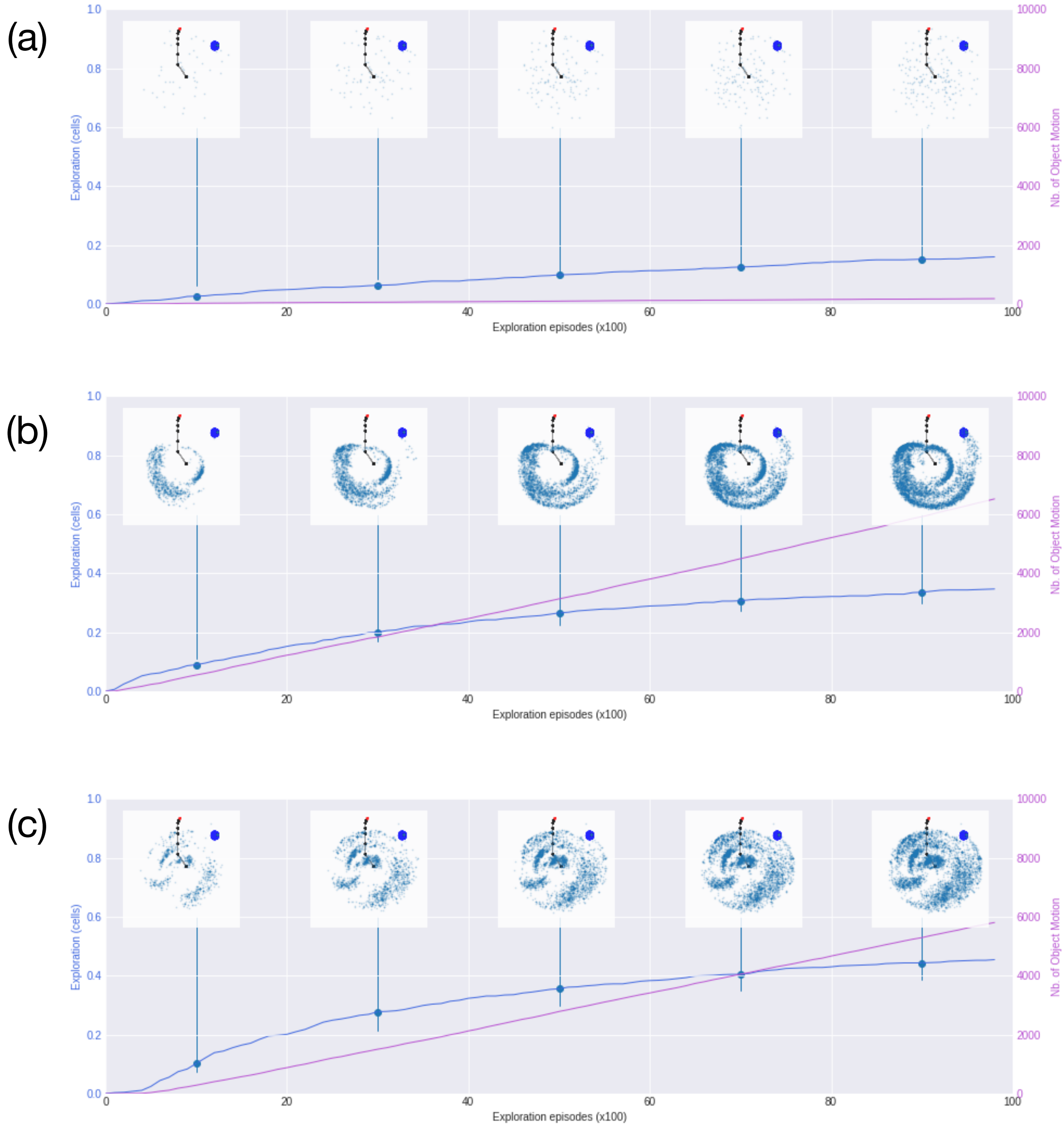

Figure S6. Examples of achieved observations together with the ratio of covered cells in the *Arm-2-Balls* environment for **RPE** (a), **RGE-EFR** (b) and **RGE-VAE** (c) exploration algorithms. The number of times the ball was effectively handled is also represented.

1.7 Exploration Curves

1.7.1 Scatter plots *Arm-2-Balls* environment

Examples of exploration curves obtained with all the exploration algorithms discussed in this paper are represented in Figure S6. Visual inspection shows that the diversity of outcomes is greater using goal exploration algorithm.

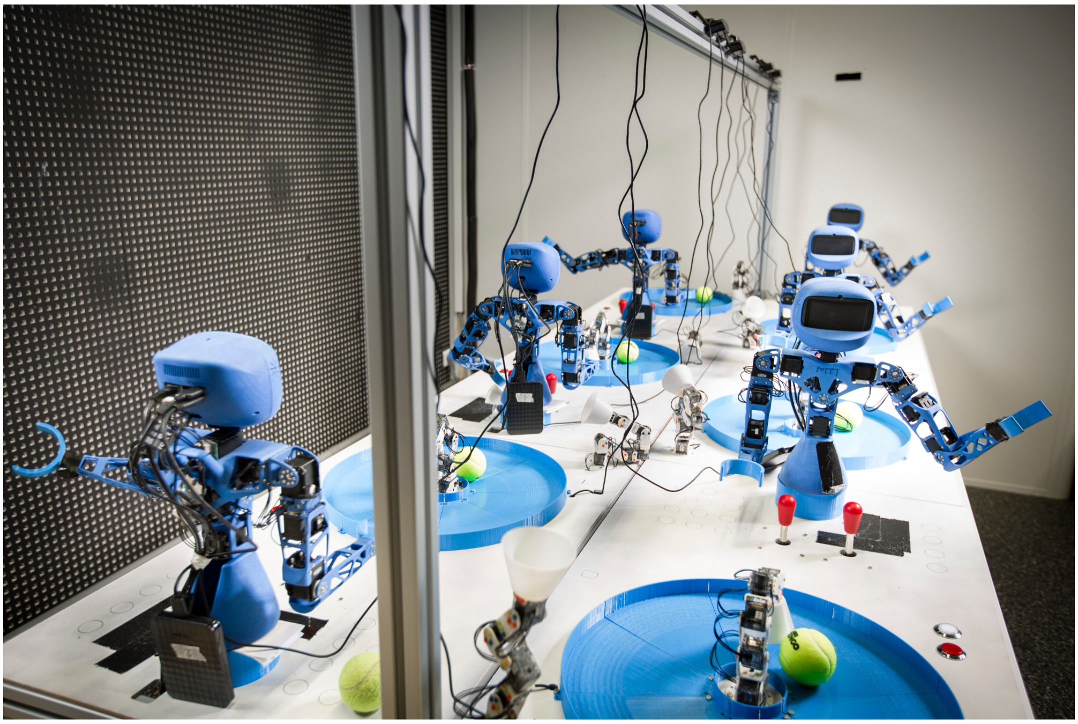

Figure S7. Experiments are performed in parallel over 6 robots. In this experiment only the 6-joints robotic arm inside the arena is used.

1.8 Experimental setup

In practice experiments are performed in parallel using multiple copies of the same experiment. A picture of the complete experimental setup is represented in Figure S7. Only the 6-joints robotic arm in the center of the arena is used in the experiments presented in this paper. Camera extracting the images are located on the bar above the setup.

REFERENCES

Baranes, A. and Oudeyer, P. Y. (2013). Active learning of inverse models with intrinsically motivated goal exploration in robots. *Robotics and Autonomous Systems* 61, 49–73. doi:10.1016/j.robot.2012.05.008

Benureau, F. C. Y. and Oudeyer, P.-Y. (2016). Behavioral Diversity Generation in Autonomous Exploration through Reuse of Past Experience. *Frontiers in Robotics and AI* 3. doi:10.3389/frobt.2016.00008

Colas, C., Fournier, P., Sigaud, O., Chetouani, M., and Oudeyer, P.-Y. (2019). Curious: Intrinsically motivated multi-task, multi-goal reinforcement learning. In *International Conference on Machine Learning (ICML)*

Forestier, S., Mollard, Y., and Oudeyer, P.-Y. (2017). Intrinsically motivated goal exploration processes with automatic curriculum learning. *arXiv preprint arXiv:1708.02190*

Forestier, S. and Oudeyer, P. Y. (2016). Modular active curiosity-driven discovery of tool use. *IEEE International Conference on Intelligent Robots and Systems* 2016-Novem, 3965–3972. doi:10.1109/IROS.2016.7759584

Higgins, I., Matthey, L., Glorot, X., Pal, A., Uria, B., Blundell, C., et al. (2016). Early Visual Concept Learning with Unsupervised Deep Learning. *arXiv preprint arXiv:1606.05579*

Higgins, I., Matthey, L., Pal, A., Burgess, C., Glorot, X., Botvinick, M., et al. (2017a). beta-VAE: Learning Basic Visual Concepts with a Constrained Variational Framework. In *ICLR*

Higgins, I., Pal, A., Rusu, A. A., Matthey, L., Burgess, C. P., Pritzel, A., et al. (2017b). DARLA: Improving Zero-Shot Transfer in Reinforcement Learning. *ICML*

Ijspeert, A. J., Nakanishi, J., Hoffmann, H., Pastor, P., and Schaal, S. (2013). Dynamical movement primitives: learning attractor models for motor behaviors. *Neural computation* 25, 328–373

Kingma, D. P. and Ba, J. L. (2015). Adam: a Method for Stochastic Optimization. *International Conference on Learning Representations*

Laversanne-Finot, A., Pere, A., and Oudeyer, P.-Y. (2018). Curiosity driven exploration of learned disentangled goal spaces. In *Proceedings of The 2nd Conference on Robot Learning* (PMLR), vol. 87 of *Proceedings of Machine Learning Research*, 487–504

Nakanishi, J., Morimoto, J., Endo, G., Cheng, G., Schaal, S., and Kawato, M. (2004). Learning from demonstration and adaptation of biped locomotion. *Robotics and autonomous systems* 47, 79–91

Pastor, P., Kalakrishnan, M., Meier, F., Stulp, F., Buchli, J., Theodorou, E., et al. (2013). From dynamic movement primitives to associative skill memories. *Robotics and Autonomous Systems* 61, 351–361

Pathak, D., Mahmoudieh, P., Luo, G., Agrawal, P., Chen, D., Shentu, Y., et al. (2018). Zero-Shot Visual Imitation. In *ICLR*. 1–12

Plappert, M., Houthooft, R., Dhariwal, P., Sidor, S., Chen, R. Y., Chen, X., et al. (2017). Parameter space noise for exploration. *arXiv preprint arXiv:1706.01905*
